# Tauroursodeoxycholic bile acid arrests axonal degeneration by inhibiting the unfolded protein response in X-linked adrenoleukodystrophy

**DOI:** 10.1007/s00401-016-1655-9

**Published:** 2016-12-21

**Authors:** Nathalie Launay, Montserrat Ruiz, Laia Grau, Francisco J. Ortega, Ekaterina V. Ilieva, Juan José Martínez, Elena Galea, Isidre Ferrer, Erwin Knecht, Aurora Pujol, Stéphane Fourcade

**Affiliations:** 1 Neurometabolic Diseases Laboratory, Institut de Neuropatologia de Bellvitge, IDIBELL, Gran Via, 199, L’Hospitalet de Llobregat, 08908 Barcelona, Spain; 2 CIBERER U759, Center for Biomedical Research on Rare Diseases, Barcelona, Spain; 3 Global data, London, UK; 4 Institute of Neurosciences, Universitat Autònoma de Barcelona, Barcelona, Spain; 5 Catalan Institution of Research and Advanced Studies (ICREA), Barcelona, Spain; 6 Institute of Neuropathology, Pathologic Anatomy Service, IDIBELL, Barcelona, Spain; 7 Department of Pathology and Experimental Therapeutics, University of Barcelona, 08908 L’Hospitalet de Llobregat, Barcelona, Spain; 8 CIBERNED, Center for Biomedical Research on Neurodegenerative Diseases, Barcelona, Spain; 9 Laboratory of Cellular Biology, Centro de Investigación Príncipe Felipe, 46012 Valencia, Spain; 10 CIBERER U721, Center for Biomedical Research on Rare Diseases, Valencia, Spain

**Keywords:** Peroxisome, ER stress, UPR, Oxidative stress, Neurodegeneration, TUDCA, Adrenoleukodystrophy

## Abstract

The activation of the highly conserved unfolded protein response (UPR) is prominent in the pathogenesis of the most prevalent neurodegenerative disorders, such as Alzheimer’s disease (AD), Parkinson’s disease (PD) and amyotrophic lateral sclerosis (ALS), which are classically characterized by an accumulation of aggregated or misfolded proteins. This activation is orchestrated by three endoplasmic reticulum (ER) stress sensors: PERK, ATF6 and IRE1. These sensors transduce signals that induce the expression of the UPR gene programme. Here, we first identified an early activator of the UPR and investigated the role of a chronically activated UPR in the pathogenesis of X-linked adrenoleukodystrophy (X-ALD), a neurometabolic disorder that is caused by ABCD1 malfunction; ABCD1 transports very long-chain fatty acids (VLCFA) into peroxisomes. The disease manifests as inflammatory demyelination in the brain or and/or degeneration of corticospinal tracts, thereby resulting in spastic paraplegia, with the accumulation of intracellular VLCFA instead of protein aggregates. Using X-ALD mouse model (*Abcd1* ^−^ and *Abcd1* ^−^ */Abcd2* ^−*/*−^ mice) and X-ALD patient’s fibroblasts and brain samples, we discovered an early engagement of the UPR. The response was characterized by the activation of the PERK and ATF6 pathways, but not the IRE1 pathway, showing a difference from the models of AD, PD or ALS. Inhibition of PERK leads to the disruption of homeostasis and increased apoptosis during ER stress induced in X-ALD fibroblasts. Redox imbalance appears to be the mechanism that initiates ER stress in X-ALD. Most importantly, we demonstrated that the bile acid tauroursodeoxycholate (TUDCA) abolishes UPR activation, which results in improvement of axonal degeneration and its associated locomotor impairment in *Abcd1* ^−^ */Abcd2* ^−*/*−^ mice. Altogether, our preclinical data provide evidence for establishing the UPR as a key drug target in the pathogenesis cascade. Our study also highlights the potential role of TUDCA as a treatment for X-ALD and other axonopathies in which similar molecular mediators are implicated.

**Electronic supplementary material:**

The online version of this article (doi:10.1007/s00401-016-1655-9) contains supplementary material, which is available to authorized users.

## Introduction

The unfolded protein response (UPR) is a cellular stress reaction of the endoplasmic reticulum (ER) that is caused by defective protein processing [36, 82, 100]. Under physiological conditions, misfolded proteins undergo the ER-associated degradation process (ERAD), whereby the ER degradation-enhancing α-mannosidase-like protein (EDEM) forms complexes with other proteins, such as the protein disulphide isomerase (PDI) and the glucose-regulated protein 78 (GRP78; also known as Bip) and glucose-regulated protein 94 (GRP94) chaperones, to guide the translocation of misfolded proteins back to the cytosol. The misfolded proteins are then polyubiquitinated for targeted degradation by the proteasome. Through this pathway, the so-called UPR is activated by an aberrant accumulation of misfolded or unfolded proteins in the ER compartment due to changes in intra-reticular calcium, altered protein glycosylation, energy deprivation, pathogen infection, expression of folding-defective proteins, or changes in redox status. The UPR is distinguished by the action of three ER-located transmembrane receptors (protein kinase RNA-like endoplasmic reticulum kinase (PERK), activating transcription factor (ATF6) and inositol requiring kinase (IRE1)), which regulate these events in concert.

Thus, in mammalian cells all three pathways, which are the so-called ER stress sensors, are involved in the UPR and have partially overlapping roles as described below [36, 82, 100]. After ER stress, unfolded proteins accumulate and PERK, along with additional proteins, attenuates mRNA translation, thereby preventing protein overload into the already stressed ER compartment. This translational attenuation is mediated by the phosphorylation of eukaryotic translation initiation factor 2 subunit α (eIF2α), which is essential for adaptation to cellular stress through the integrated stress response (ISR) process. The phosphorylation of eIF2α enables the preferential translation of UPR-dependent genes, such as the transcription factor ATF4, which drives the transcription of several critical genes, including the pro-apoptotic factor CHOP [36, 82, 100].

ATF6 is a transcription factor that translocates to the Golgi compartment upon ER stress, where it is cleaved [105]. Cleaved ATF6 then enters the nucleus to activate target genes, such as *GRP78*, *GRP94*, *PDI* and *CHOP*.

Upon activation, IRE1, which is a transmembrane kinase/endonuclease, initiates the splicing of the X-box-binding protein 1 (*XBP1*) mRNA. Spliced *XBP1* (*XBP1s*) mRNA leads to the transcription of several genes that are involved in the UPR and ERAD to restore protein homeostasis and promote cytoprotection [36, 82, 100]. In conclusion, the first outcome of the UPR is protective as it helps maintain homeostasis. However, if the stress is not successfully resolved, the UPR can finally trigger apoptosis. The same pathways can be anti- or pro-apoptotic depending on the intensity, duration and cellular context of the UPR activation [36, 44, 78, 82, 100].

Unfolded protein response activation has classically been associated with several neurodegenerative and metabolic diseases that are characterized by protein aggregates, including Alzheimer’s disease (AD) [41, 42, 82], Huntington’s disease (HD) [95], amyotrophic lateral sclerosis (ALS) [81], Parkinson’s disease (PD) [82], Pick’s disease [47], and argyrophilic grain disease [46]. Despite several studies that support the interconnected nature of UPR activation, only a few reports have addressed the role of the ER and UPR in diseases that are driven by redox and lipid dyshomeostasis instead of protein misfolding [61, 91]. We therefore sought to explore the role of ER stress in a paradigmatic disease of oxidative stress and lipid rather than protein accumulation: X-linked adrenoleukodystrophy (X-ALD: McKusick no. 300100) [28, 57].

X-ALD is a rare, fatal, neurometabolic disease that is characterized by a striking variation in clinical symptoms, even within the same family. The two main neurological phenotypes include (i) the devastating childhood cerebral form (CCALD), which progresses rapidly and is associated with rampant inflammatory demyelination in the brain, and the more common phenotype of (ii) slowly progressing adult-onset adrenomyeloneuropathy (AMN), which manifests as a distal axonopathy of corticospinal tracts, thereby leading to spastic paraparesis with peripheral neuropathy in some cases [26, 66]. Approximately 35% of AMN patients develop cerebral demyelination (cAMN) and share the same poor prognosis as children with cerebral ALD. All patients with X-ALD have mutations in the *ABCD1* gene in Xq28, which encodes the peroxisomal adrenoleukodystrophy protein (ALDP or ABCD1) [26, 66]; ALDP transports very long-chain fatty acids (VLCFA) or VLCFA–CoA esters into the peroxisome for degradation by β-oxidation [93]. Therapeutic options are scarce, and when diagnosed early, the cerebral forms of the disease are only adequately treatable with a bone marrow transplant [1] or, recently, haematopoietic stem cell gene therapy [9]. However, no pharmacological treatment has been proven to be beneficial for either form of the disease [6].

The mouse model of X-ALD, the *Abcd1* ^−^ mouse, develops late-onset axonopathy that resembles the most frequent X-ALD phenotype, AMN [75, 76]. Using this mouse model and patient samples, studies by our laboratory and others have revealed that VLCFA-induced oxidative stress is an early, key pathogenic factor in X-ALD [29, 31, 35, 57, 73, 74, 85, 94], although the exact mechanisms by which oxidative stress causes neurodegeneration in X-ALD are not entirely clear.

Oxidative stress and lipid alterations are highly intertwined with ER stress [61, 91, 97, 98]; we thus posited that UPR activation may have a role in the pathogenesis of X-ALD. To test this hypothesis, we determined whether the UPR (i) is activated in brain samples of patients who suffered from the cerebral forms of X-ALD (CCALD and cAMN), the spinal cord from the mouse model of X-ALD and in human X-ALD fibroblasts and (ii) is a causative factor of axonal degeneration, which we determined by assessing the effects of the ER stress inhibitor tauroursodeoxycholic acid (TUDCA) on the late-onset axonopathic phenotype that is exhibited in the X-ALD (*Abcd1* ^−^ */Abcd2* ^−*/*−^) mouse model.

## Materials and methods

### Human samples

Brain tissue samples from X-ALD patients and age-matched controls were obtained from the NIH NeuroBioBank: details are in the supplemental data (Supplemental Table S1).

Primary human fibroblasts were collected from healthy individuals and AMN patients according to the IDIBELL guidelines for sampling, including informed consent from the persons involved or their representatives. Fibroblasts were prepared from skin biopsies (Supplemental Table S2).

### X-ALD mice

Two X-ALD mouse models were used in this study. The first model, the *Abcd1* ^−^ mouse, is used to characterize the biochemical signs as it resembles adult AMN in humans [59, 76]. These mice exhibit metabolic signs of pathology, including oxidative stress [29] and altered energy homeostasis [34]; however, the first clinical signs of AMN (axonopathy and locomotor impairment) appear at 20 months [76]. The second mouse model has a double gene knockout of both the *Abcd1* and *Abcd2* transporters (*Abcd1* ^−^ */Abcd2* ^−*/*−^) and is a more suitable model for assaying therapeutic strategies than *Abcd1* ^−^ mice. Indeed, compared with *Abcd1* ^−^ mice, *Abcd1* ^−^ */Abcd2* ^−*/*−^mice present enhanced VLCFA accumulation in the spinal cord [75], higher levels of oxidative damage to proteins [31], and a more severe AMN-like pathology with an earlier onset at 12 months of age [30, 55, 62, 65, 75].

Male mice used for experiments were of a pure C57BL/6J background. Generation and genotyping for *Abcd1* ^−^ and *Abcd1* ^−^ */Abcd2* ^−*/*−^ mice have been previously described [59, 75, 76].

For the *antioxidant treatment*, wild type (WT) (*n* = 10) and *Abcd1* ^−^ mice (*n* = 20) were separated into three different groups at 8 months of age [WT (*n* = 10), *Abcd1* ^−^ (*n* = 10) and *Abcd1* ^−^ mice treated with a cocktail of antioxidants [Vitamin E (1000 iu/kg) and α-lipoic acid (0.5%) in AIN-76A chow (Ssniff) and NAC (1%) in water] for 4 months (*Abcd1* ^−^ + Aox) (*n* = 10)] [34, 56, 57]. At 12 months of age, mice were killed, and tissue samples were collected and snap-frozen in liquid nitrogen.

For the *TUDCA treatment*, WT (*n* = 10) and *Abcd1* ^−^ mice (*n* = 20) were separated into three different groups at 9 months of age [WT (*n* = 10), *Abcd1* ^−^ (*n* = 10) and *Abcd1* ^−^ mice treated with the bile acid TUDCA (Prodotti Chimici e Alimentari SpA, Italy) for 3 months (*Abcd1* ^−^ + TUDCA) (*n* = 10)]. TUDCA was mixed into AIN-76A chow (Ssniff) at 0.4% w/w. In addition, WT (*n* = 15) and *Abcd1* ^−^ */Abcd2* ^−*/*−^ mice (*n* = 30) were separated into three different groups at 13 months of age [WT (*n* = 15), *Abcd1* ^−^ */Abcd2* ^−*/*−^ (*n* = 15) and *Abcd1* ^−^ */Abcd2* ^−*/*−^ mice treated with TUDCA (0.4% w/w) for 4 months (*Abcd1* ^−^ */Abcd2* ^−*/*−^ + TUDCA) (*n* = 15)]. TUDCA had no effect on weight or food intake under either treatment protocol. The dose and duration of TUDCA, whose pharmacokinetics have been extensively studied in rodents [14], was based on pilot studies using APP/PS1 mice and other models [11, 67].

After 4 months of treatment, locomotor experiments were performed for three weeks, one test a week, in order not to exhaust the mice. Animals were maintained under treatment during this period and were killed and processed at the end for immunohistology. For behavioural testing, assessment of hindlimb clasping was done by suspending WT and *Abcd1* ^−^ */Abcd2* ^−/−^ mice from their tails until they reached a vertical position, allowing the mice to grab the grill of the lid of the cage with the forelimbs, as adapted from Dumser et al. [19]. The hindlimb reflex was analysed for 10 s in three consecutive trials separated by 5-min rest. The hindlimb reflex was scored as described in Supplemental Table S3. The treadmill and bar cross experiments were performed exactly as previously described [57, 64]. Mock- and drug-treated mice were simultaneously tested using these three locomotor tests (clasping, treadmill and bar cross) for both genotype and treatment in a blinded manner.

All methods employed in this study were in accordance with the Guide for the Care and Use of Laboratory Animals published by the US National Institutes of Health (NIH publication No. 85-23, revised 1996) and the ethical committees of IDIBELL and the Generalitat de Catalunya.

### Antibodies and reagents

Detailed information on antibodies is summarized in Supplemental Table S4. Hexacosanoic acid (C26:0), propidium iodide (PI) and tunicamycin (TM) were purchased from Sigma (St. Louis, MO). The PERK inhibitor GSK2606414 was purchased from Calbiochem.

### Immunofluorescence and immunohistochemistry

For all experiments, spinal cords were embedded in paraffin, and serial sections (4 µm thick) were cut in a transversal or longitudinal (1 cm long) plane after perfusion with 4% paraformaldehyde.

For immunofluorescence studies, spinal cords from 3- and 12-month-old wild type and *Abcd1* ^−^ mice were incubated with PDI, GRP78 or GFAP antibodies. Then, confocal images were acquired using a Leica TCS SL laser scanning confocal spectral microscope (Leica Microsystems Heidelberg GmbH, Mannheim, Germany), and images were analysed with ImageJ 1.39 u (NIH, USA). Details of the immunofluorescence processing can be found in the supplemental experimental procedures.

Immunohistological (IHC) studies performed in WT, *Abcd1* ^−^ */Abcd2* ^−*/*−^ and *Abcd1* ^−^ */Abcd2* ^−*/*−^ mice treated with TUDCA were carried out with the avidin–biotin peroxidase method, as reported earlier [65].

The results were expressed as the mean ± SD.

### Reverse transcription (RT)-PCR analysis

Quantitative RT-PCR analysis was performed as previously described [65]. The expression of the genes of interest was analysed by Q-PCR using TaqMan^®^ Gene Expression Assays (Applied Biosystems). Relative quantitation was carried out using the ‘Delta–Delta Ct’ (ΔΔCt) method with RPL0 as the endogenous control. Transcript quantification was performed in duplicate for each sample [65].

### Cell culture and treatment

Primary human fibroblasts were cultured in DMEM (containing 10% foetal bovine serum, 100 U/mL penicillin and 100 µg streptomycin) at 37 °C in humidified 5% CO_2_/95% air. Unless otherwise stated, experiments were carried out with cells at 80% confluence. Cells were pretreated 1 h with 120 nM of PERK inhibitor GSK2606414 and then exposed to 2 μg/mL of tunicamycin (TM) for 48 h or 72 h. Cell lines were used on passages 12–18.

For viability experiments, cells were incubated with 10 μg/mL propidium iodide for 10 min in the dark after treatment and then analysed with an Agilent 2100 Bioanalyzer (Gallios Beckman Coulter). The apoptotic cells were quantified with FlowJo software.

### Statistical analysis

Statistical significance was assessed using the Student’s *t* test whenever two groups were compared. When analysing multiple groups, we used a one-way ANOVA and Tukey’s post hoc test to determine statistical significance. Data are presented as the mean ± SD (*P < 0.05; **P < 0.01; ***P < 0.001).

### Supplemental experimental procedures

Details of *XBP1* mRNA processing, immunoblots and nuclear fractionation experiments can be found in the supplemental experimental procedures.

## Results

In this work, we analysed three different types of samples associated with X-ALD, including necropsy samples of brain white matter from patients with cerebral inflammatory disease (CCALD and cAMN), patient fibroblasts, and spinal cords from the X-ALD mouse model (*Abcd1* ^−^ and *Abcd1* ^−^ */Abcd2* ^−*/*−^ mice). We examined the following markers of UPR activation: (i) the induction of the ER stress sensors PERK, ATF6 and IRE1 and (ii) the expression of the UPR responsive proteins ATF4, CHOP, GADD34, GRP78, GRP94 and PDI.

### The UPR is induced in the brain samples of patients with X-ALD

**Fig. 1:**
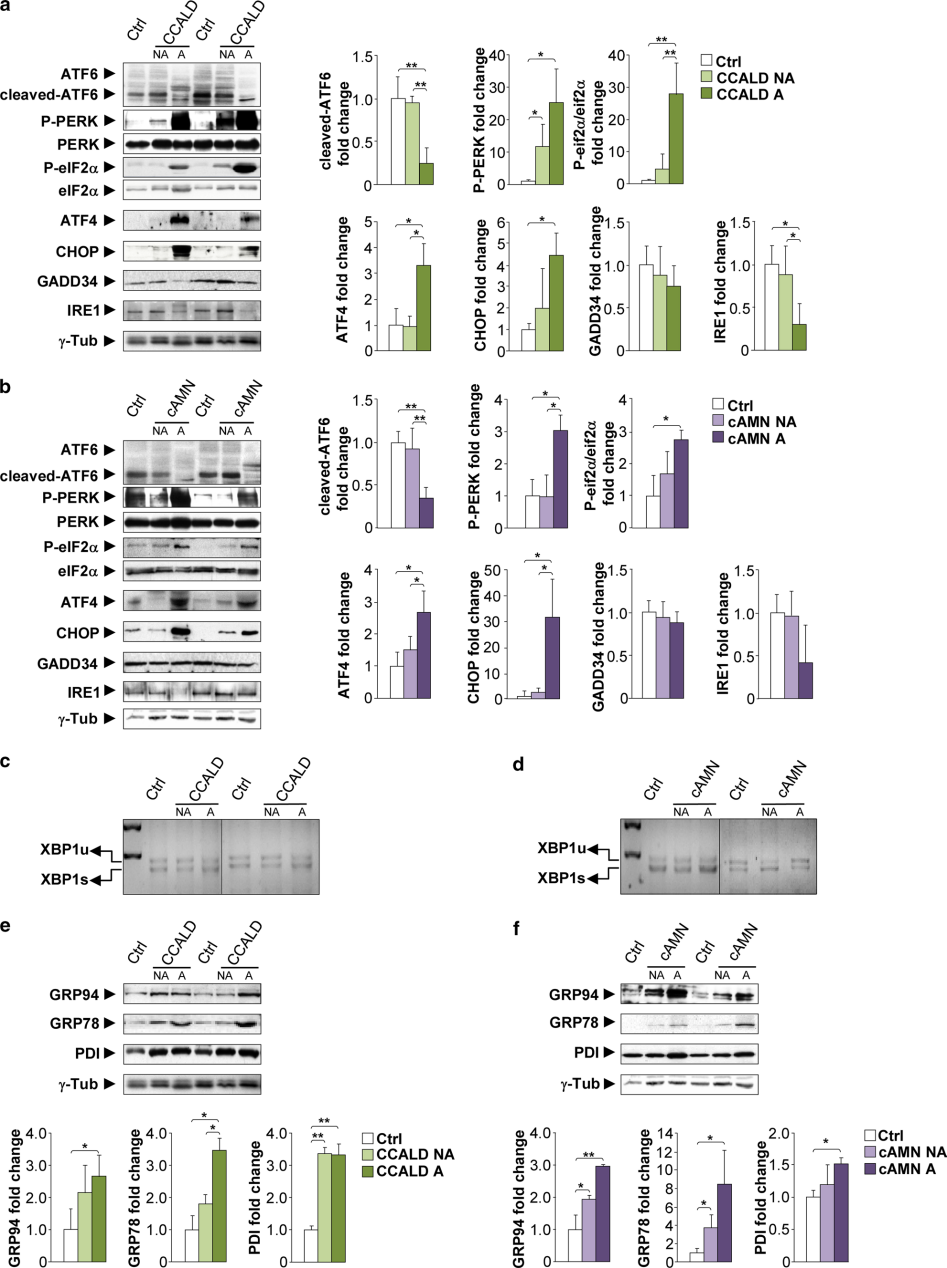
**a**,** b** Induction of the UPR in CCALD and cAMN patient brains. Representative immunoblots for full-length ATF6, cleaved-ATF6, total PERK, phosphorylated PERK (P-PERK), eIF2α, phosphorylated-eIF2α (P-eIF2α), ATF4, CHOP, GADD34 and IRE1 levels in total lysates from controls (Ctrl) and in normal-appearing (NA) and affected (A) white matter from CCALD (**a**) and cAMN patients (**b**). *XBP1* mRNA splicing analysis by RT-PCR in Ctrl samples and NA and A white matter from CCALD (**c**) and cAMN patients (**d**). Unspliced- and spliced-*XBP1* mRNA corresponds to *XBP1u* mRNA and *XBP1s* mRNA, respectively. Representative immunoblots for GRP78, GRP94, and PDI levels in Ctrl samples and NA and A white matter from CCALD (**e**) and cAMN patients (**f**). Protein levels were normalized relative to γ-tubulin (γ-Tub). The histograms on the *right* (**a**, **b**) and *below* (**e**, **f**) show the ratio and the protein levels relative to control. All values are expressed as the mean ± SD (*n* = 5 by genotype and condition in **a**–**f**; *P < 0.05 and **P < 0. 01, one-way ANOVA followed by Tukey’s HSD post hoc test for **a**, **b**, **e** and **f**)

Analysis of PERK phosphorylation and the P-eIF2α/eIF2α ratio showed an increase in PERK and eIF2α phosphorylation levels in the affected areas of CCALD and cAMN patients (Fig. 1a, b). In addition, we found a significant up-regulation of ATF4 levels in the affected areas of CCALD and cAMN patients, which correlated with an induction of CHOP in the affected areas of both phenotypes (Fig. 1a, b). However, for the same samples, no change was detected in the levels of GADD34, another target gene of ATF4 (Fig. 1a, b).

We assessed ATF6 activation by measuring the liberation of its cleaved fragment. We showed lower cleaved-ATF6 levels in the affected areas of the CCALD and cAMN brains than in the normal-appearing areas and matched healthy individuals (Fig. 1a, b). Finally, immunoblot experiments showed that the levels of the IRE1 transducer were decreased in the affected zones of CCALD and cAMN brains (Fig. 1a, b). In addition, both unspliced and spliced *XBP1* mRNA levels were similar in control, non-affected and affected white matter, indicating that the IRE1 pathway was not overactivated in X-ALD patients (Fig. 1c, d).

In affected white matter samples of CCALD and cAMN patients, ATF6 mRNA and protein shared the same pattern of repression, which was correlated with decreased expression of *GRP78*, *GRP94, EDEM2* and *HERPUD1* mRNA levels, (Supplemental Fig. S1a, b). However, protein expression of GRP78, GRP94 and PDI was induced in the same samples (Fig. 1e, f). That may be due to a lack of degradation of GRP78 and GRP94, which may undergo posttranslational modifications such as S-nitrosylation, as shown for PDI and GRP78 [23, 25, 26]. Under normal conditions, these proteins would be degraded by the proteasome or autophagy routes, which may not occur in X-ALD as both systems are malfunctioning, as we have previously reported [8, 9].

In conclusion, the PERK pathway but not the IRE1 or ATF6 pathways are activated in the affected white matter of CCALD and cAMN patients. In the spinal cords from cAMN patients, we obtained similar results regarding PERK activation and for the ATF6 pathway which was not modified (Supplemental Fig. S2a, b).

### Pathway-specific regulation of the UPR in *Abcd1*^−^ mice

**Fig. 2:**
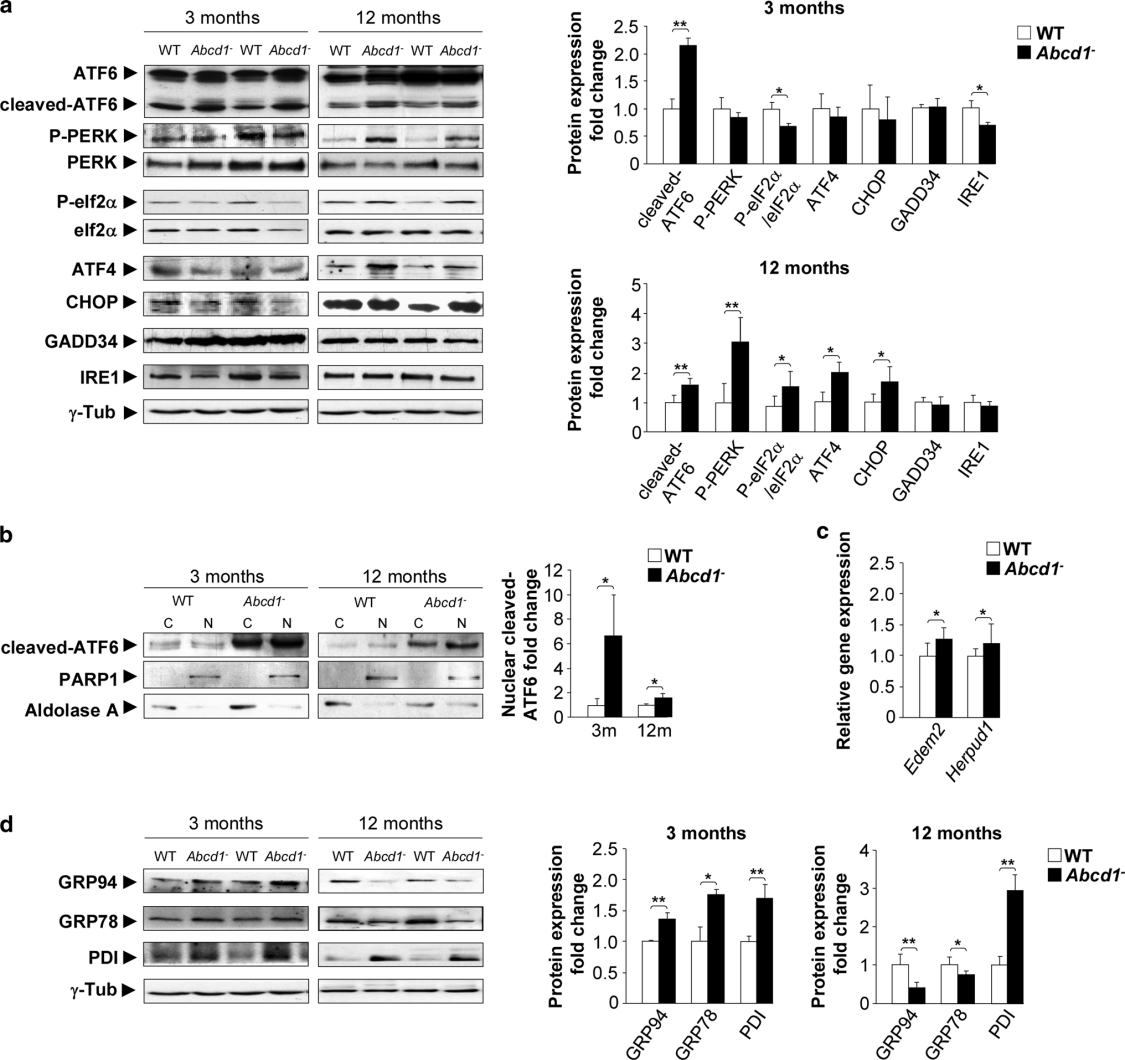
UPR induction in the X-ALD mouse model. **a** Representative immunoblots of ER stress sensors full-length ATF6, cleaved-ATF6, total PERK, P-PERK, eIF2α, P-eIF2α, ATF4, CHOP, GADD34 and IRE1 in the spinal cord tissue of *Abcd1* ^−^ mice and age-matched wild type (WT) mice at 3 and 12 months of age. The histograms on the *right* show the cleaved-ATF6, P-PERK, ATF4, CHOP, GADD34 and IRE1 levels normalized relative to γ-Tub and the P-PERK/PERK and the P-eIF2α/eIF2α ratios relative to their respective WT values. **b** The nuclear localization of cleaved-ATF6 in WT and *Abcd1* ^−^ mice at 3 and 12 months of age. PARP1 was used as the control for the nuclear fraction (N) and aldolase A was used for the cytoplasmic fraction (C). The histograms on the *right* show the cleaved-ATF6 levels relative to WT values in the nuclear fractions. **c** Real-time RT-PCR analyses of *Edem2* and *Herpud1* mRNA at 12 months in *Abcd1* ^−^ mouse spinal cords. **d** Immunoblots of GRP94 and GRP78 chaperones and PDI in the spinal cord tissue of *Abcd1* ^−^ mice and age-matched wild type (WT) mice 3 and 12 months of age. The histograms on the *right* show the GRP94 and GRP78 chaperones and PDI levels normalized to WT mice and normalized relative to the γ-Tub. Values are expressed as the mean ± SD (*n* = 10 samples per genotype and condition in **a**, **c** and **d**; *n* = 6 samples per genotype and condition in **b**; *P < 0.05, **P < 0.01 and ***P < 0.001, Student’s *t* test)

We measured the activation of ER sensors in the spinal cords from *Abcd1* ^−^ mice early in adulthood, at three months and at 12 months of age, well prior to disease onset, which occurs at 20 months. PERK phosphorylation and the P-eIF2α/eIF2α ratio were increased in X-ALD mice at 12 months but not at three months of age. This PERK pathway activation was confirmed by enhanced ATF4 and CHOP levels in *Abcd1* ^−^ spinal cord tissue starting at 12 months. Finally, in agreement with the observations in the brain samples, we could not detect differences in the GADD34 levels at either three or 12 months of age (Fig. 2a). In contrast, here we detected increased levels of cleaved-ATF6 at three, and to a smaller extent, 12 months (Fig. 2a). To confirm the activation of the ATF6 pathway, we analysed the translocation of cleaved-ATF6 into the nucleus and found that cleaved-ATF6 was increased in *Abcd1* ^−^ mouse spinal cord tissue at three, and to a lesser extent, 12 months (Fig. 2b). Since ATF6 also activates genes involved in the ERAD, we measured the expression of *Edem2* and *Herpud1* mRNA at 12 months of age. Indeed, expression of these ERAD genes appeared to be induced (Fig. 2c). The IRE1 transducer was not activated at three or 12 months, as demonstrated by the lack of spliced XBP1 (mRNA and protein) in *Abcd1* ^−^ mouse spinal cord tissue (Supplemental Fig. S3a, b).

Taken together, these data indicate that the ER stress response is an early phenomenon in the X-ALD mouse model, with a strong activation of the ATF6 pathway at three months of age and a later activation of the PERK and ATF6 but not IRE1 pathways.

### Time-dependent expression of chaperones and PDI in motor neurons and astrocytes of *Abcd1*^−^ mice

**Fig. 3:**
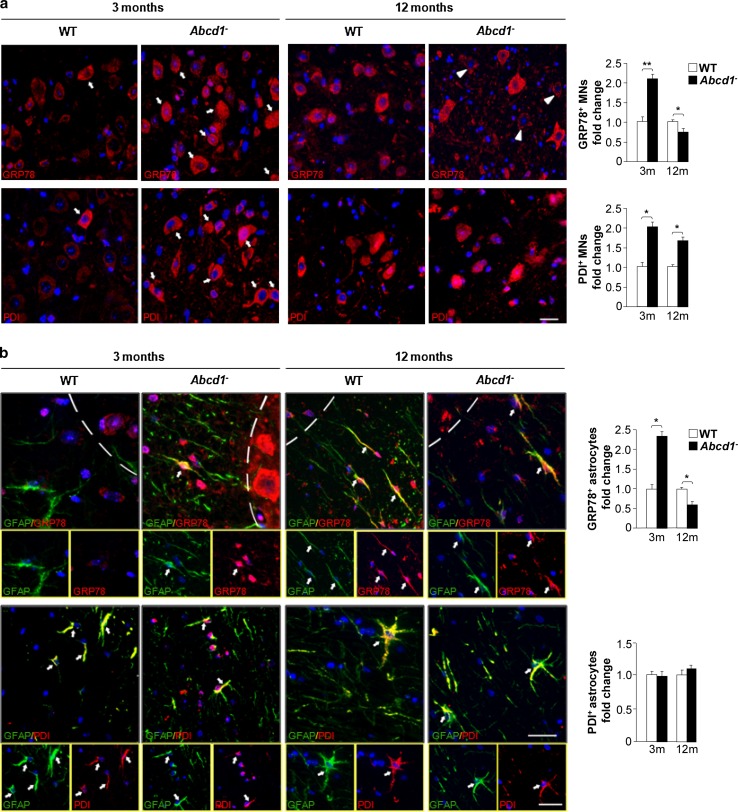
The UPR is primarily induced in motor neurons and astrocytes during X-ALD pathogenesis. **a** Immunofluorescence of GRP78 and PDI in spinal cord sections of WT and *Abcd1* ^−^ mice 3 and 12 months of age. *Arrows* indicate high amounts of GRP78 and PDI in motor neurons; *Arrowheads* denote motor neurons with low amounts of GRP78. Nuclei were counterstained with DAPI. *Scale bar* 50 μm. **b** Dual immunolabelling of astrocytes (GFAP; *green*) with GRP78 or PDI (*red*) in spinal cord sections of WT and *Abcd1* ^−^ mice 3 and 12 months of age. Positive cells are labelled with *arrows*, and the *dashed-line* stands for the limit between the grey- and white matter. Nuclei were counterstained with DAPI. The *small panel below* shows some double-positive astrocytes. *Scale bar* 25 μm. The histogram on the *right* represents the quantification of GRP78 and PDI fluorescence intensity normalized to WT mice in motor neurons (MNs) (**a**) and astrocytes (**b**). Values are expressed as the mean ± SD (*n* = 4 samples per genotype and condition in **a** and **b**; *P < 0.05, **P < 0.01 and ***P < 0.001, Student’s *t* test)

The expression of the mRNA and protein of the chaperones GRP78, GRP94 and PDI was up-regulated in the total extracts from the spinal cord of 3-month-old *Abcd1* ^−^ mice. At 12 months of age, the increase in PDI persisted (protein and mRNA), whereas GRP78 and GRP94 protein levels were down-regulated in the mutant mice compared with WT littermates (Fig. 2d). Moreover, *Grp78* and *Grp94* mRNA levels were not altered and increased, respectively, at this age in *Abcd1* ^−^ mice (Supplemental Fig. S3c). To gain insight into the cell-type specificity of the observed response, we performed immunofluorescence in spinal cord sections. Prominent GRP78 and PDI immunostaining was observed in motor neurons of the *Abcd1* ^−^ mouse spinal cord at three months compared with that in the WT mice (Fig. 3a), which is consistent with the results obtained by western blot (Fig. 2d). We also detected an up-regulation of GRP78 expression but not an increase in PDI staining in astrocytes of *Abcd1* ^−^ mice at three months of age (Fig. 3b). At 12 months of age, we observed results similar to those obtained by western blot (i.e. decreased expression of GRP78 in both motor neurons and astrocytes and an induction of PDI staining in motor neurons from *Abcd1* ^−^ mice) (Fig. 3a, b).

### UPR induction in fibroblasts from X-ALD patients

The fibroblasts of X-ALD patients are a good surrogate cell model for dissecting disease mechanisms, as they recapitulate the main X-ALD hallmarks: (i) accumulation of VLCFA [101], (ii) higher production of free radicals of mitochondrial origin [58], (iii) loss of energetic homeostasis [34], and (iv) altered proteostasis [55, 56]. Using this cell system, we sought to determine whether an induction of ER stress was detected in fibroblasts from X-ALD patients.

**Fig. 4:**
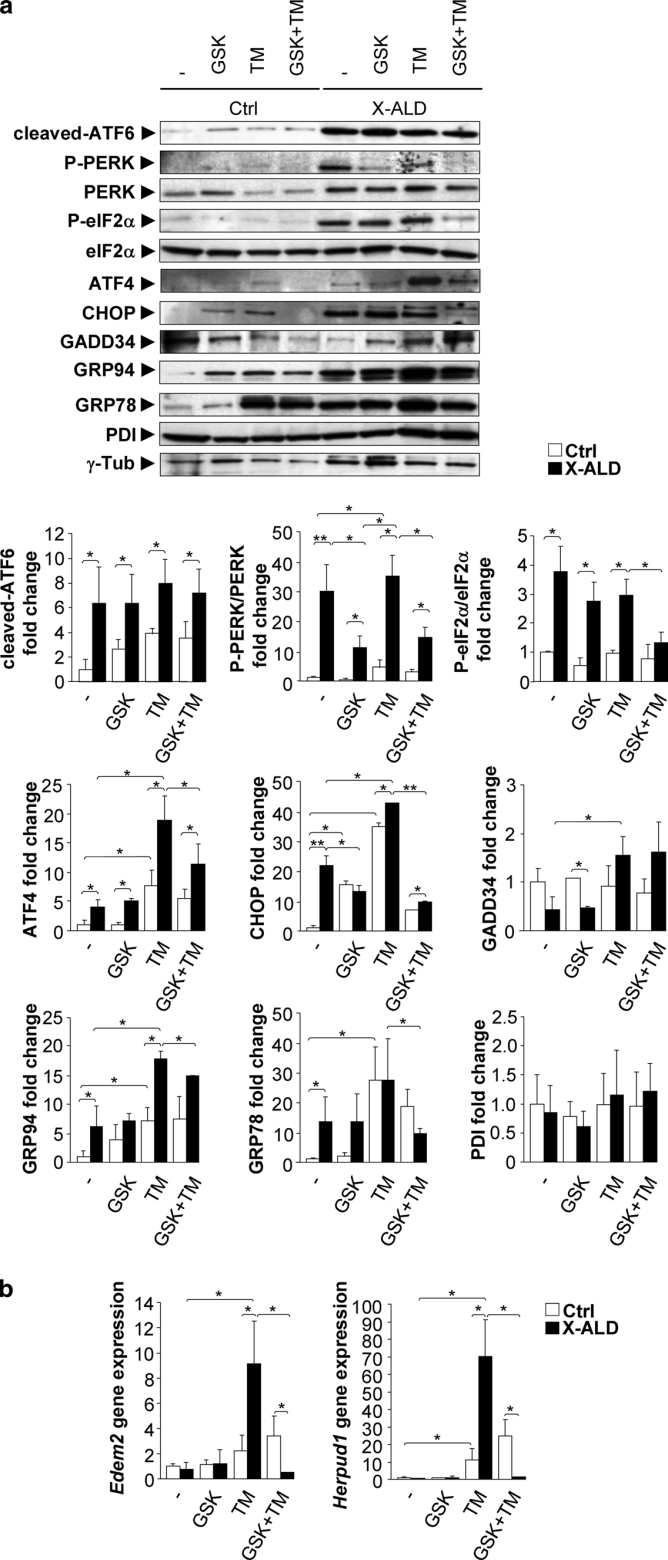
Inhibition of PERK disrupts the UPR and renders the X-ALD fibroblasts more susceptible to ER stress. **a** Representative immunoblots for cleaved-ATF6, total PERK, P-PERK, total eIF2α, P-eIF2α, ATF4, CHOP, GADD34, GRP78, GRP94 and PDI levels in control (Ctrl) and X-ALD human fibroblasts pretreated with or without the PERK inhibitor GSK2606414 (GSK, 120 nM) for 1 h and then exposed to tunicamycin (TM, 2 µg/mL) for 48 h. Protein levels were normalized relative to γ-tubulin (γ-Tub). The histograms *below* show the cleaved-ATF6, P-PERK, P-eIF2α, ATF4, CHOP, GADD34, GRP94, GRP78 and PDI levels normalized relative to γ-Tub and the P-PERK/PERK and P-eIF2α/eIF2α ratios relative to their respective WT values. **b** Real-time RT-PCR analyses of *Edem2* and *Herpud1* mRNA levels in Ctrl and X-ALD human fibroblasts pretreated with or without the PERK inhibitor GSK2606414 (GSK; 120 nM) for 1 h and then exposed to tunicamycin (TM, 2 µg/mL) for 48 h. All values are expressed as the mean ± SD (*n* = 4 by genotype and condition; *P < 0.05 and **P < 0. 01, one-way ANOVA followed by Tukey’s HSD post hoc test)

At baseline, we found increases in the levels of cleaved-ATF6, ATF4, CHOP, and PERK and eIF2α phosphorylation, thereby mimicking the pattern of ER stress sensor activation in the spinal cords of 12-month-old *Abcd1* ^−^ mice (Fig. 4a). Moreover, we detected an increase in the protein levels of GRP94 and GRP78 in X-ALD fibroblasts compared to that in the control cells at baseline (Fig. 4a).

### Inhibition of PERK disrupts the UPR and renders the X-ALD fibroblasts more susceptible to ER stress

To determine whether UPR activation was protective or detrimental in the X-ALD fibroblasts, we targeted the UPR machinery using tunicamycin (TM), which blocks N-linked glycosylation, inducing protein misfolding and the UPR. After a 48-h incubation with 2 µg/mL TM, we detected a slight but significant 10% increase in cell death in the X-ALD fibroblasts compared to that in the controls (Supplemental Fig. S4). In these conditions, we found that the PERK pathway was induced in the X-ALD fibroblasts as they presented significantly higher ATF4, CHOP and GADD34 protein levels than baseline (Fig. 4a). Of note, the mRNA levels of *Edem2* and *Herpud1* were strongly increased in X-ALD fibroblasts upon tunicamycin treatment, suggesting that the ERAD machinery was more reactive to TM-induced stress (Fig. 4b). In the control fibroblasts, we found a similar pattern but a less intense response.

Since the PERK pathway appeared to be a pivotal player in the TM-induced UPR activation, we assessed the effects of the specific PERK inhibitor GSK2606414 on the observed UPR-induced cell death. We found that the PERK inhibitor induced a more premature cell death in X-ALD than control fibroblasts following 48 h with 2 µg/mL TM (25% for X-ALD and 12% for controls), while GSK2606414 alone did not have any effect on cell death (Supplemental Fig. S4). At basal levels, GSK2606414 significantly decreased PERK phosphorylation and CHOP levels in X-ALD fibroblasts in comparison with control fibroblasts.

Following 2 µg/mL TM plus 120 nM GSK2606414 treatment for 48 h, we found a significant decrease in P-PERK, P-eIF2α, ATF4 and CHOP levels in X-ALD fibroblasts. In contrast to control fibroblasts, the only significant decrease was in the expression of CHOP (Fig. 4a). Interestingly, the GRP94 and GRP78 protein levels and *Edem2* and *Herpud1* mRNA levels were also substantially reduced with PERK inhibition in the X-ALD fibroblasts following 48 h TM treatment (Fig. 4a, b). These data suggest that PERK is a major participant in the transcriptional control of ATF6 and many UPR target genes in response to tunicamycin treatment in X-ALD fibroblasts, including those involved in protein folding, such as GRP94 or GRP78 and ERAD factors such as EDEM2 or HERPUD1.

Altogether, these results suggest that PERK inhibition blocks the induction of eIF2α phosphorylation and the translational control arm of the UPR, which leads to disruption of homeostasis and increased apoptosis during ER stress induced in X-ALD fibroblasts. Theses results were in accordance with the study by Teske et al. [88], showing that the PERK pathway facilitates both the synthesis of ATF6 and trafficking of ATF6 from the ER to the Golgi for intramembrane proteolysis and activation of ATF6. As a consequence, PERK loss of function could significantly reduce both the translational and transcriptional phases of the UPR, leading to reduced protein chaperone expression and enhanced apoptosis.

### Antioxidant treatment prevents ER stress and UPR induction in *Abcd1*^−^ mice

**Fig. 5:**
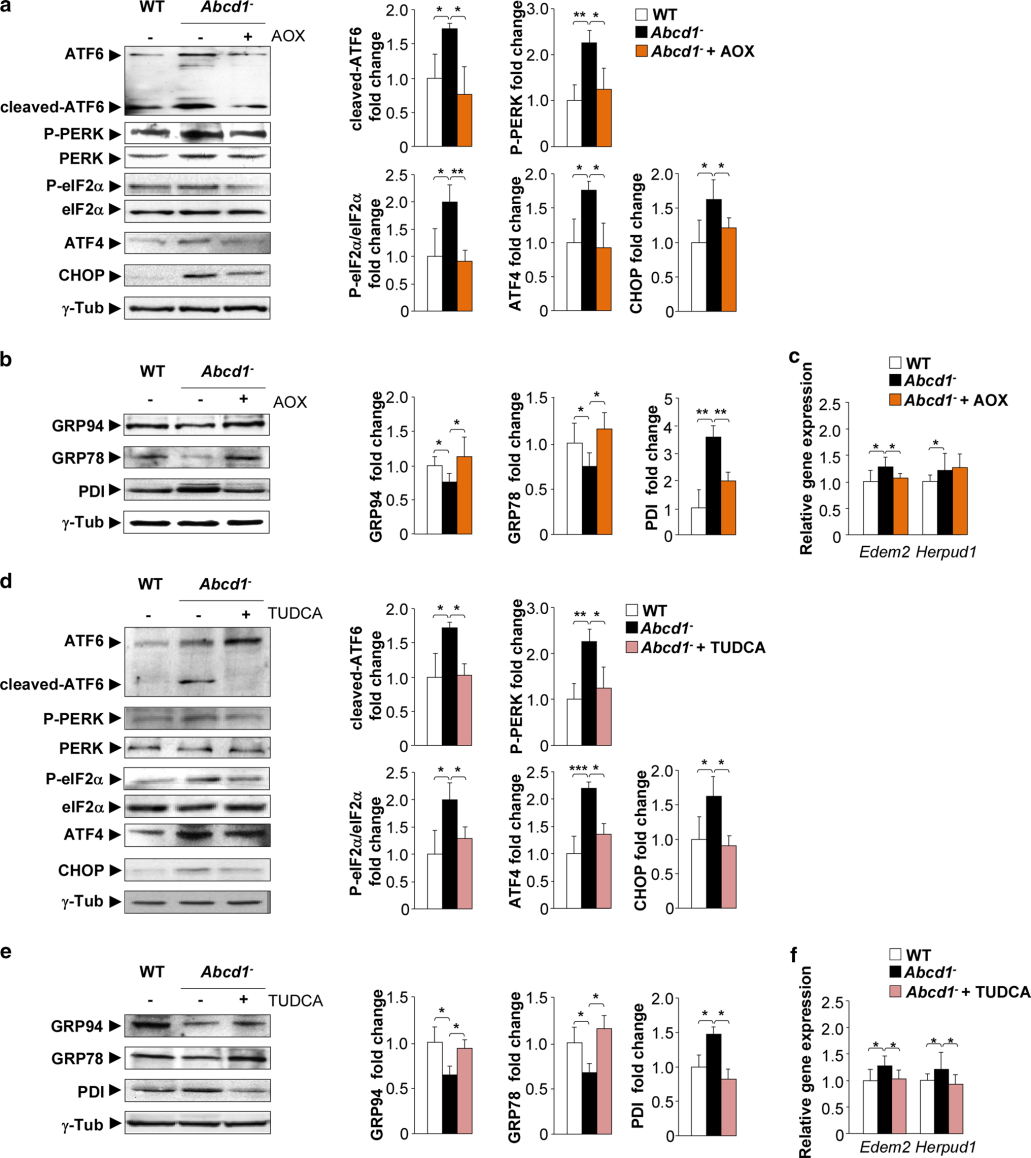
Antioxidant and TUDCA treatments prevent UPR activation in the X-ALD mouse model. **a**, **d** Representative immunoblots for full-length ATF6, cleaved-ATF6, total PERK, P-PERK, total eIF2α, P-eIF2α, ATF4 and CHOP in the spinal cord tissue of WT mice (WT), *Abcd1* ^−^ mice (*Abcd1* ^−^) and **a** antioxidant-treated (*Abcd1* ^−^ + AOX) or **d** TUDCA-treated (*Abcd1* ^−^ + TUDCA) *Abcd1* ^−^ mice at 12 months of age. GRP78, GRP94 and PDI levels were analysed in the spinal cords of WT, *Abcd1* ^−^ and *Abcd1* ^−^ + AOX mice (**b**) and *Abcd1* ^−^ + TUDCA mice (**e**) at 12 months of age. Real-time RT-PCR analyses of *Edem2* and *Herpud1* in the spinal cord tissue of WT mice (WT), *Abcd1* ^−^ mice (*Abcd1* ^−^) and **c** antioxidant-treated (*Abcd1* ^−^ + AOX) or **f** TUDCA-treated (*Abcd1* ^−^ + TUDCA) *Abcd1* ^−^ mice at 12 months of age. In (**a**, **b** and **d**, **e**), the histograms on the *right* show normalized UPR marker levels relative to those in untreated WT mice. All values are expressed as the mean ± SD (*n* = 10 by genotype and condition in **a**–**d**; **P < 0.01 and ***P < 0.001, one-way ANOVA followed by Tukey’s HSD post hoc test)

We previously demonstrated that VLCFA excess produces free radicals, and as a consequence, early oxidative damage is present in the spinal cords of *Abcd1* ^−^ mice [29]. A combination of vitamin E, α-lipoic acid and NAC efficiently reduced ROS production in vitro, reversed oxidative damage to proteins and DNA in spinal cords, and arrested axonal degeneration and disability in X-ALD mice [57]. To determine whether UPR is regulated by redox imbalance in vivo, we treated the presymptomatic *Abcd1* ^−^ mice at eight months of age with the above-mentioned combination of antioxidants for four months. The antioxidant treatment normalized the levels of cleaved-ATF6, phosphorylated PERK, phosphorylated eIF2α, ATF4 and CHOP (Fig. 5a), GRP94, GRP78 and PDI (Fig. 5b) and *Edem2* mRNA (Fig. 5c) in *Abcd1* ^−^ mice. These data demonstrate that oxidative stress is an upstream promoter of UPR activation in X-ALD mice.

### The bile acid TUDCA reduces the UPR activation in *Abcd1*^−^ mice

Chemical chaperones have previously been shown to be effective at reducing ER stress [70, 72]. TUDCA, which is a well-known member of this chemical chaperone family, is a unique bile acid that acts as a potent anti-apoptotic agent. Previous studies have shown its involvement as a neuroprotective agent in neurodegenerative diseases where the UPR is considered to be a trigger of pathogenic mechanisms, such as AD [17], PD [10] and ALS [106]. Accordingly, *Abcd1* ^−^ mice were fed a diet that was supplemented with 0.4% (wt/wt) TUDCA for three months starting at nine months of age. Then, we assessed the protein levels of several UPR responsive genes and ER stress sensors that were previously identified as being dysregulated in the *Abcd1* ^−^ mice. The levels of cleaved-ATF6, P-PERK, P-eIF2α/eIF2α, ATF4 and CHOP (Fig. 5d), the levels of GRP78 and GRP94 and PDI (Fig. 5e) and the mRNA levels of *Edem2* and *Herpud1* (Fig. 5f) were normalized in *Abcd1* ^−^ mice following exposure to TUDCA.

### TUDCA halts axonal degeneration and locomotor deficit progression in *Abcd1*^−^*/Abcd2*^−/−^ mice

**Fig. 6:**
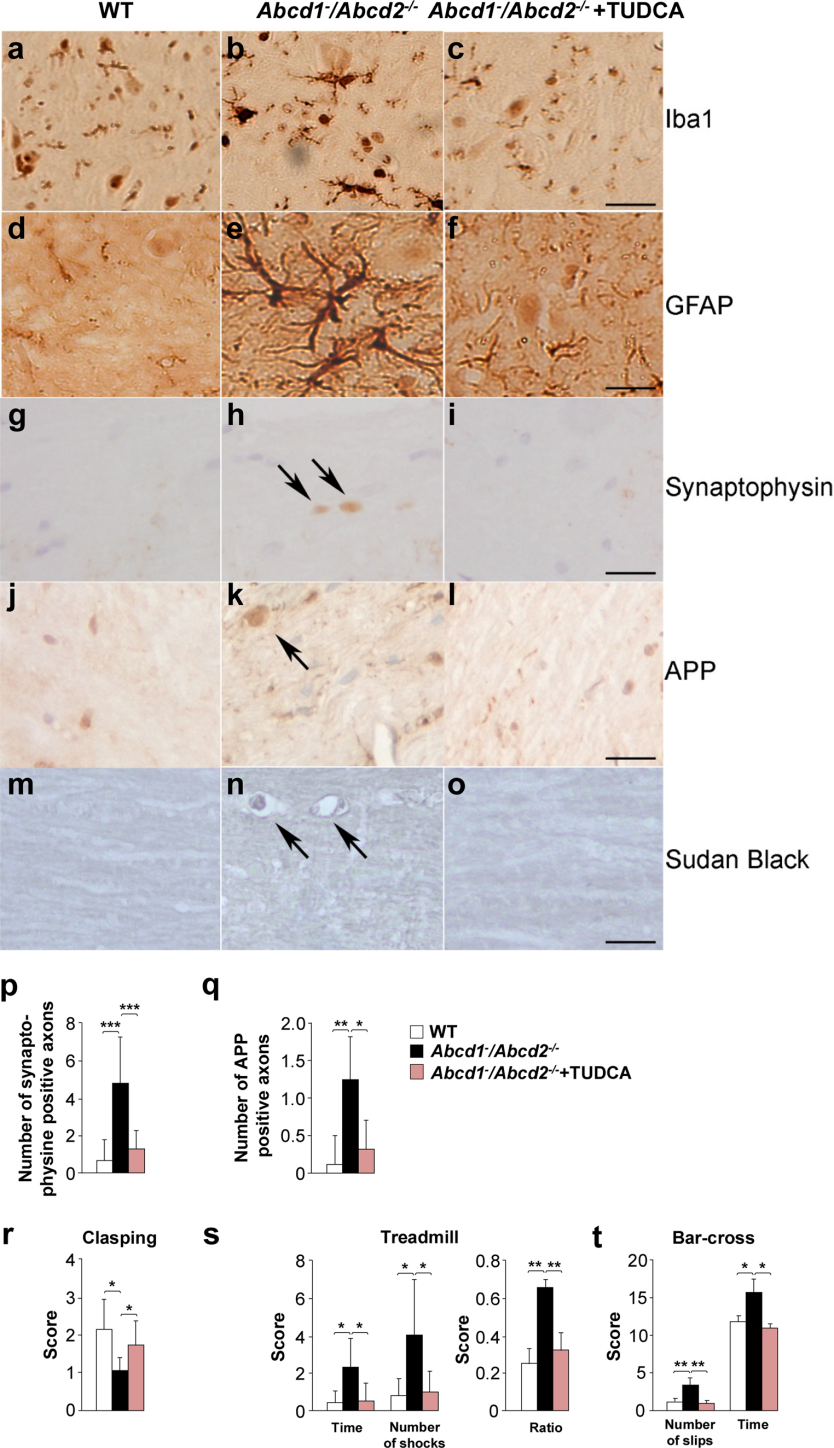
**a**–**q** TUDCA halts axonal degeneration and locomotor disability in *Abcd1* ^−^ */Abcd2* ^−/−^ mice. Immunohistological analysis of axonal pathologies performed on WT, *Abcd1* ^−^ */Abcd2* ^−*/*−^ and TUDCA-treated *Abcd1* ^−^ */Abcd2* ^−*/*−^ mice (*Abcd1* ^−^ */Abcd2* ^−*/*−^ + TUDCA) of 18 months of age. Spinal cord immunohistological sections were processed for **a**–**c** Iba1, **d**–**f** GFAP, **g**–**i** synaptophysin, **j**–**l** APP and **m**–**o** Sudan black. Representative images for WT (**a**, **d**, **g**, **j** and **m)**, *Abcd1* ^−^ */Abcd2* ^−/−^ (**b**, **e**, **h**, **k**, and **n)**, and *Abcd1* ^−^ */Abcd2* ^−/−^ + TUDCA (**c**, **f**, **i**, **l** and **o)** mice are shown. *Bars* 25 µm. The quantification of synaptophysin (**p**) and APP (**q**) in 1-cm-long longitudinal sections of the dorsal spinal cord in WT, *Abcd1* ^−^ */Abcd2* ^−*/*−^ and *Abcd1* ^−^ */Abcd2* ^−*/*−^ + TUDCA mice at 18 m of age (*n* = 5 mice per genotype and condition). The number of abnormal specific profiles was counted at every ten sections for each stain. At least five sections of the spinal cord were analysed per animal and per stain. Clasping (**r**), treadmill (**s**) and bar cross (**t**) tests were conducted on WT, *Abcd1* ^−^ */Abcd2* ^−/−^ and TUDCA-treated *Abcd1* ^−^ */Abcd2* ^−/−^ mice (*Abcd1* ^−^ */Abcd2* ^−/−^ + TUDCA) 17 months of age. **r** The best performance score of each animal was used for statistical analysis [19]. **s** The latency to falling from the belt (time of shocks), the number of shocks received and the ratio were computed after 5 min. **t** The time spent to cross the bar and the numbers of slips of the hind limbs were quantified. Values are expressed as the mean ± SD (*n* = 5 per condition in **a**–**q**; *n* = 15 per condition in **r**–**t**; *P < 0.05, **P < 0.01 and ***P < 0.001, one-way ANOVA followed by Tukey’s HSD post hoc test)

We first observed that the UPR was also activated in *Abcd1* ^−^ */Abcd2* ^−*/*−^ mice, following a similar pattern than in *Abcd1* ^−^ mice (Supplemental Fig. S5). To assess the preclinical potential of the treatment, we treated with TUDCA cohorts of *Abcd1* ^−^ */Abcd2* ^−/−^ right at disease onset, starting at 13 months of age until 17 months of age. As observed in *Abcd1* ^−^ mice (Fig. 5d, e), TUDCA is able to inhibit UPR activation in this model (Supplemental Fig. S5). We then evaluated axonal degeneration by semi-quantifying the accumulation of the axonal damage markers synaptophysin and APP, the scattered myelin debris with Sudan black staining, the number of reactive microglia and reactive astrocytes and its activated-state morphology. Therefore, we observed that these measures were strikingly reduced to control levels following TUDCA treatment (Fig. 6a–q; Supplemental Table S5).

We next evaluated locomotor deficits in *Abcd1* ^−^ */Abcd2* ^−/−^ mice using clasping [19], treadmill and bar cross experiments [55, 57, 64, 65] after TUDCA treatment. In the clasping test, the best performance score of each animal was used for statistical analysis. Compared with WT mice, *Abcd1* ^−^ */Abcd2* ^−/−^ mice presented a lower score, thereby demonstrating a locomotor deficit (Fig. 6r). Remarkably, TUDCA treatment normalized this score. In the treadmill experiment, the double-knockout mice required longer shock times and more frequent shocks to run at belt speed than the WT mice, as described earlier [55, 57, 64, 65]. After four months of treatment, this ratio was indistinguishable from the WT ratio (Fig. 6s). Finally, in the bar cross experiments, double-knockout mutants often failed to maintain their balance and displayed a greater tendency of the hind limbs to slip off of the bar and longer latencies to reaching the platform at the opposite end of the bar [55, 57, 64, 65]. The number of slips and the time necessary to cross the bar were also normalized following TUDCA treatment (Fig. 6t). Overall, these data show that TUDCA treatment arrested the progression of the locomotor deficits that occur in *Abcd1* ^−^ */Abcd2* ^−*/*−^ mice. Altogether, these results established a direct link between UPR activation, axonal degeneration and locomotor deficits in X-ALD.

## Discussion

### An ER stress signature in peroxisomal disorders?

Peroxisomes are oxidative organelles present in all tissues and organisms [83, 84, 102, 103] and are involved in several anabolic and catabolic reactions, including the following: the degradation of long- and very long-chain fatty acids (LCFA, VLCFA (C ≥ 22:0), respectively) by α- or β-oxidation and hydrogen peroxide detoxification and the synthesis of bile acids, plasmalogens and essential polyunsaturated fatty acids. The loss or malfunction of peroxisomes is the cause of more than 20 fatal inherited conditions, which are classified into two main groups: (i) peroxisome biogenesis (PBD) and (ii) single peroxisomal enzyme deficiencies. Mutations in peroxisomal proteins that are essential for biogenesis and membrane protein import (called peroxins or *PEX* genes) invariably lead to PBD, with Zellweger syndrome as the most severe manifestation [3, 16, 83, 102, 103], (see also http://www.peroxisomedb.org).

Morphological abnormalities of the ER compartment have been described in peroxisomal mutants, including *Pex5* ^−*/*−^ [18] and *Abcd2* ^−/−^ mice [27]. Interestingly, previous studies using *Pex2* ^−*/*−^ as a model of PBD [53, 54] or *Acox1* ^−/−^ as a model of single peroxisomal enzyme deficiency [45] have shown that ER stress is induced. Indeed, the ER stress response in the *Pex2* ^−*/*−^ liver revealed a predominant activation of specific UPR branches (i.e. the PERK-ATF4 pathway), with increased mRNA expression of CHOP in conjunction with a lack or paucity of IRE1 activation and the absence of subsequent *XBP1* mRNA splicing [54]. The panoply of metabolic abnormalities in *Pex2* ^−*/*−^ liver tissue [24, 99] includes the perturbed flux of mevalonate, bile acids, plasmalogens, docosahexaenoic acid (DHA, C22:6ω3) and VLCFA. Moreover, the same type of UPR activation (excluding IRE1) occurs in *Acox1* ^−/−^ liver tissue [45], in which the first enzyme of the peroxisomal β-oxidation, the so-called fatty acyl-CoA oxidase 1, is inactive; thus, only the levels of DHA and VLCFA but not plasmalogens or bile acid are altered [2, 45, 48]. In *Abcd1* ^−^ mice with peroxisomal malfunctions that are largely related to defective import-induced excessive VLCFA, we observed a similar UPR pattern with predominant activation of the PERK-ATF4 pathway and a blunted IRE1/Xbp1 response at 12 months of age.

This finding is in contrast with the phenotype in models of other leukodystrophies, such as vanishing white matter disease and Pelizaeus–Merzbacher disease (PMD), in which all three branches of the UPR are activated [86, 92]. The same observations occur in models of ALS [51], AD [52], tauopathies [39, 52] and synucleinopathies [12, 13].

### Contribution of peroxisomal malfunction to UPR activation in neurodegenerative disorders

The ER and peroxisomes share not only a common evolutionary history and ontogeny [40, 84, 87] but also many intertwined metabolic pathways, particularly the synthesis of the fatty acids that form the lateral chains of complex lipids, such as sphingolipids, phospholipids and plasmalogens, which are main components of membrane lipid bilayers [102]. For instance, the bile acids and DHA are partially synthesized in the ER before entering the peroxisome for the last steps of their synthesis. In addition, VLCFA are elongated from C16:0 in the ER [50, 68], and subsequently imported by ABCD1 and ABCD2 transporters into peroxisomes for degradation via β-oxidation [30, 93]. The last steps of plasmalogen biosynthesis occur in the ER, but the required intermediates are previously synthesized in the peroxisome [25, 102]. As a consequence, peroxisomal deficiency results in an accumulation of toxic bile acid precursors (tri-hydroxycholestanic acid (THCA) and di-hydroxycholestanic acid (DHCA)) [7, 25] (Supplemental Fig. S6) as well as VLCFA [7], with reduced levels of protective, antioxidant compounds, such as DHA and plasmalogens, in several types of tissues from PBD patients [7].

These observations suggest that UPR activation could be a direct consequence of the unbalanced lipid metabolism that results from peroxisome malfunction.

**Fig. 7:**
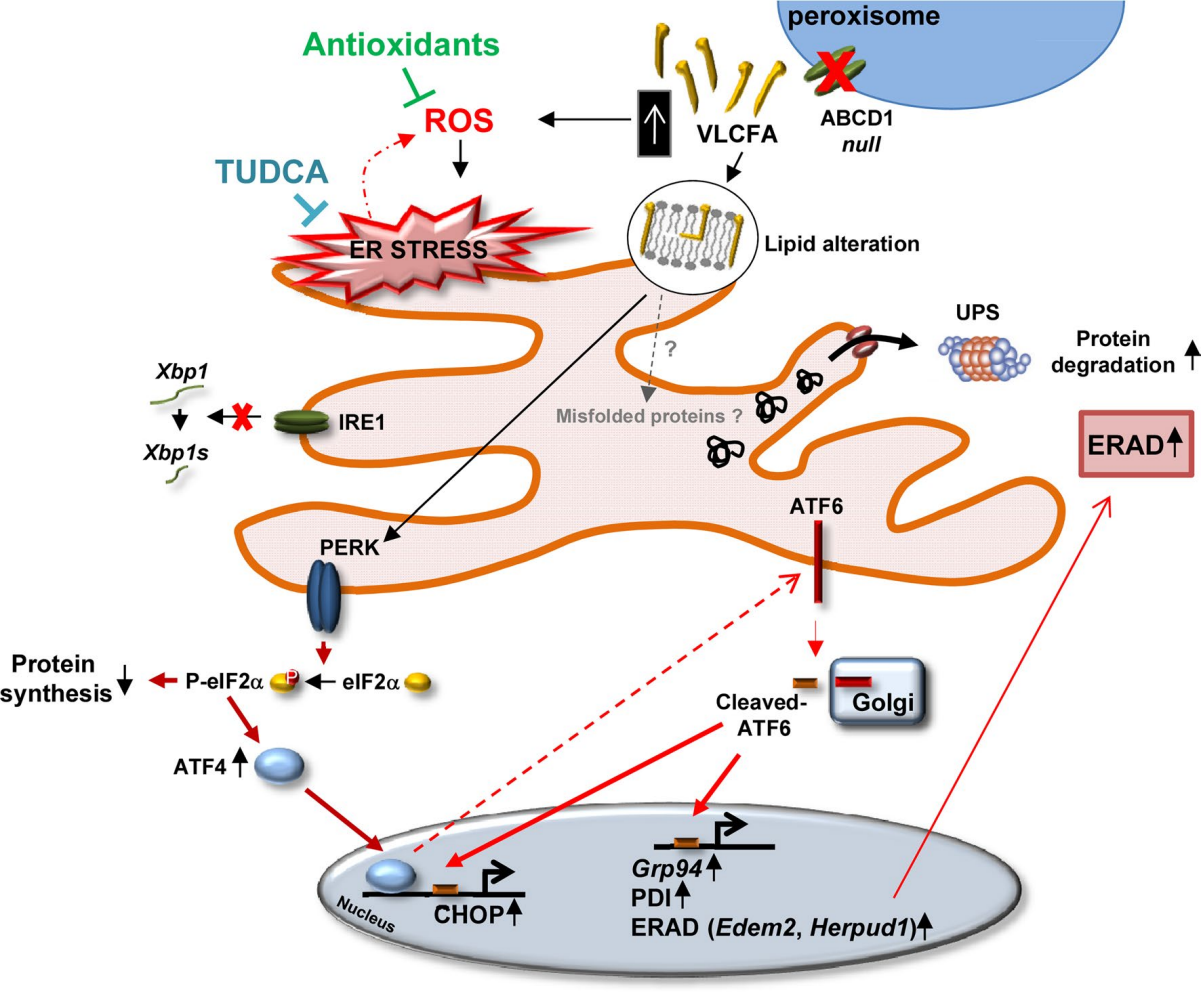
Mechanisms of UPR activation in the spinal cord from an X-ALD mouse model. Representation of the ER stress response following excess VLCFA due to the lack of ABCD1 function in the X-ALD mouse model. Prior to disease onset (at 12 months), the PERK/P-eIF2α/ATF4 pathway is activated. It is central for translational control but also for the activation of ATF6 during ER stress, and as a consequence, is critical for the transcription of its target genes, including those involved in protein folding or the ERAD. Oxidative stress produced by excess VLCFA and possibly also ER membrane lipid perturbations induced by accumulation of these fatty acids could induce PERK activation independently of unfolded protein formation. This would constitute an adaptive mechanism, allowing the cell to engage ER folding and the ERAD machinery response. Both antioxidant and TUDCA treatments of X-ALD mice prevent ER stress activation and halt subsequent axonal neurodegeneration

This is supported by in vivo and in vitro studies that show that the activation of the UPR is directly correlated to a high concentration of saturated phospholipids in ER membranes, in the absence of protein aggregates in the ER lumen, as is the case for the deletion of the mediator subunit mdt-15, a regulator of the transcription of genes involved in fatty acid (FA) metabolism [43]. We thus posit that VLCFA excess may modify the fluidity of the ER lipid bilayer, as shown in model membranes [38, 104]. In X-ALD, this perturbation due to VLCFA excess has recently been speculated to occur in the inner mitochondrial membrane, leading to ROS production [58]. Furthermore, the elongation of VLCFA, which is located in the ER, is increased in X-ALD fibroblasts [50, 68], perhaps contributing to the UPR. In other cases, lipid disequilibrium within the ER membrane could influence the quantity and trafficking of misfolded proteins in the ER lumen, leading indirectly to the activation of UPR transducers [33, 97]. All in all, and in the absence of direct proof of protein aggregates in the ER lumen, we posit that ER membrane lipid perturbation induced by VLCFA accumulation leads to UPR transducer activation, allowing the cell to adapt the ER folding and ERAD machinery igniting an “anticipatory ER stress” response [80, 96] (Fig. 7).

A modification of the ER membrane fluidity by fatty acids is of direct relevance to diseases that are associated with increases in saturated fatty acids, such as non-alcoholic fatty liver [8, 77] or type 2 diabetes mellitus [70], which also show ER stress in the absence of misfolded proteins.

Regarding the timing of the observed responses, we observed that in X-ALD and the models of PDB or single peroxisomal enzyme deficiencies, UPR activation occured early in life [23, 45, 53]. In contrast, UPR activation is not an early event in AD [52], Tau disease [39, 52], PD [12, 13] or ALS [51]. We thus hypothesize that late UPR activation, such as that for AD or PD when compared with that for peroxisomal disorders may be related to the phenomenon of deteriorating peroxisomal fitness over time, as observed during ageing and neurodegenerative disorders [5, 21, 32, 63, 89]. Altogether, these data suggest that an altered peroxisomal function and subsequent lipid metabolism unbalance are key contributors to ER stress, both in rare and common neurodegenerative disorders.

### Bile acids in the treatment of neurodegenerative disorders?

The relevance of bile acids for brain function is illustrated by the observation of large amounts of several intermediates of bile acid biosynthesis in the cerebrospinal fluid [69] (Supplemental Fig. S6). Three of them (glycocholate, glycodeoxycholate, and glycochenodeoxycholate) were elevated in the plasma of AD patients [90], and other compounds of this route were found to be altered in X-ALD adult patients. 5β-cholestane-3α,7α,12α-triol, cholate and glycolate are accumulated in the plasma, whereas 5β-cholestane-3α,7α,12α,26-tetrol is increased in peripheral blood mononuclear cells from AMN patients [79]. *Pex2* ^−/−^ mice also showed an accumulation of the toxic THCA and DHCA in liver tissue, which was concomitant with ER stress [49, 53].

UDCA [15] and TUDCA [70, 72] are hydrophilic bile acids that have been used to alleviate ER stress. TUDCA is of particular interest because its levels are lowered in *Pex2* ^−*/*−^ mice [25], thereby suggesting a contribution to ER stress. Indeed, a diet that is enriched in cholic acid and UDCA was able to improve the dendritic arborization of cerebellar Purkinje cells in vivo [22], while also increasing both UDCA and TUDCA levels [49]. Moreover, the chronic administration of UDCA and TUDCA is able to modify the lipid and fatty acid composition of the ER membrane in the rat liver [4]. Treatment with TUDCA has also been shown to ameliorate ER stress in AD [17] and PD mouse models [10]. Altogether, these data suggest that UPR activation may be the result of an alteration in ER membrane fluidity, as noted above, which may be alleviated by replenishing TUDCA levels. We thus posit that levels of bile acids, including TUDCA and fatty acids, are important in ER stress-related disorders and that it is advisable to include drugs that seek to restore their normal levels when managing these diseases.

Although a systematic comparison of neurodegenerative diseases that highlights therapeutically relevant similarities and differences is still lacking, our data reinforce the notion that ER dysfunction is a pivotal, actionable target for treating neurodegenerative diseases [82]. Interestingly, here we demonstrated that the treatment of X-ALD mice with the ER stress inhibitor TUDCA reduced the progression of axonal degeneration and locomotor disabilities (Fig. 6). This finding is highly relevant because it provides a feasible therapeutic avenue for correcting peroxisomal disorders and neurodegenerative diseases. Indeed, TUDCA has outstanding in vivo safety profiles, is able to cross the blood–brain barrier, and has been approved by the U.S. Food and Drug Administration for clinical use [37]. Clinical trials for ALS and HD have recently been conducted and have yielded promising results regarding safety and efficacy (NCT00877604; NCT00514774) [20, 60, 71]. Thus, we believe that for diseases in which ER stress is an early factor in the pathogenetic cascade, the chances of success may be even greater.

## Electronic supplementary material

Below is the link to the electronic supplementary material.

Supplementary material 1 (DOCX 29 kb)

Supplementary material 2 (PDF 56 kb) Fig S1. Downregulation of ATF6 and its targets in affected areas of patient brains. (**a-b**) Real-time RT-PCR analyses of *ATF6*, *GRP78*, *GRP94*, *PDI*, *EDEM2* and *HERPUD1* mRNA in control (Ctrl) samples and in normal-appearing (NA) and affected (A) white matter from (**a**) CCALD and (**b**) cAMN patients. Values are expressed as the mean ± SD (n=5 samples per genotype; *P<0.05, **P<0.01 and ***P<0.001, one-way ANOVA followed by Tukey’s HSD *post hoc* test)

Supplementary material 3 (PDF 145 kb) Fig. S2. Induction of UPR in the spinal cord of cAMN patients. **(a)** Representative immunoblots for ATF6, PERK, phosphorylated PERK (P-PERK), eIF2α, phosphorylated-eIF2α (P-eIF2α), ATF4, CHOP and GADD34 levels in the spinal cord from control (Ctrl) and cAMN patients. (**b**) Representative immunoblots for GRP78, GRP94, and PDI levels in the spinal cord from Ctrl and cAMN patients. Protein levels are normalized to γ-tubulin (γ-Tub) levels. The histograms on the right show the ratio and the protein levels relative to control. All values are expressed as the mean ± SD (n=7 by genotype and condition in **a**-**b**; *P<0.05 and **P<0. 01, one-way ANOVA followed by Tukey’s HSD *post hoc* test for **a** and **b**)

Supplementary material 4 (PDF 122 kb) Fig. S3 (**a**) *Xbp1* cDNA PCR products were cut by PstI (PstI+), producing either two products of 291-bp and 183-bp from the native unspliced form of *Xbp1* cDNA; or an uncut product of 448-bp from the spliced *Xbp1* cDNA. Unspliced-*Xbp1* mRNA: “*Xbp1u*” mRNA; Spliced-*Xbp1* mRNA: “*Xbp1s*” mRNA. (**b**) Representative immunoblots of unspliced XBP1 proteins in spinal cords from 3- and 12-month-old *Abcd1* ^-^ mice and age-matched WT mice. The histogram on the right shows the quantification of XBP1u protein normalized to WT mice. **(c)** Real-time RT-PCR analyses of *Grp78*, *Grp94* and *Pdi* at 3 and 12 months in *Abcd1* ^-^ mouse spinal cords. Values are expressed as the mean ± SD (n= 6 samples per genotype; *P<0.05, **P<0.01 and ***P<0.001, Student’s t test)

Supplementary material 5 (PDF 723 kb) Fig. S4 X-ALD fibroblasts are more sensitive to tunicamycin upon PERK inhibition. **(a)** Control and X-ALD fibroblasts were pretreated with or without the PERK inhibitor GSK2606414 (GSK; 120 nM) for 1 h and then exposed to tunicamycin (TM, 2 µg/mL) for 48 h. Pictures of control and X-ALD fibroblasts were obtained **(a),** and cell death was measured using flow cytometry **(b)**. Values are expressed as the mean ± SD (n=4 by genotype and condition; *P<0.05, **P<0.01 and ***P<0.001, one-way ANOVA followed by Tukey’s HSD *post hoc* test)

Supplementary material 6 (PDF 162 kb) Fig. S5: UPR induction in the *Abcd1* ^-^/*Abcd2* ^*-/-*^mice. **(a**) Representative immunoblots of ER stress sensors ATF6, cleaved-ATF6, P-PERK/PERK and P-eIF2α/eIF2α ratios, ATF4, CHOP and GADD34 in the spinal cord tissue of wild type (WT), *Abcd1* ^-^/*Abcd2* ^*-/-*^ and TUDCA-treated (*Abcd1* ^-^/*Abcd2* ^*-/-*^+ TUDCA) *Abcd1* ^-^/*Abcd2* ^*-/-*^mice 18 months of age. The histograms on the right show the cleaved-ATF6, P-PERK, P-eIF2α, ATF4, CHOP and GADD34 levels normalized relative to γ-Tub and the P-PERK/PERK and P-eIF2α/eIF2α ratios relative to their respective WT values. (**c**) GRP78, GRP94 and PDI levels were analysed in the spinal cords of WT, *Abcd1* ^-^/*Abcd2* ^*-/-*^ and *Abcd1* ^-^/*Abcd2* ^*-/-*^ + TUDCA mice 18 months of age. In (**a and b**), the histograms on the right show normalized UPR marker levels relative to those in untreated WT mice. All values are expressed as the mean ± SD (n=8 by genotype and condition in **a-b**; **P<0.01 and ***P<0.001, one-way ANOVA followed by Tukey’s HSD *post hoc* test)

Supplementary material 7 (PDF 144 kb) Fig. S6 Simplified schema of bile acid biosynthesis focused on molecules mentioned in this study and cellular compartments where the biosynthesis is carried out

Supplementary material 8 (PDF 81 kb) Table S1 Description of human brain samples

Supplementary material 9 (PDF 77 kb) Table S2 Description of human X-ALD fibroblasts

Supplementary material 10 (PDF 107 kb) Table S3 Scaled score corresponding to hindlimb clasping behaviour

Supplementary material 11 (PDF 85 kb) Table S4 List of antibodies

Supplementary material 12 (PDF 77 kb) Table S5 Summary of the main pathological findings in transversal or longitudinal (1 cm long) sections of the dorsal spinal cord in WT, *Abcd1* ^-^ */Abcd2* ^-/-^, *Abcd1* ^-^ */Abcd2* ^-/-^ + TUDCA mice at 18m of age (n=5 mice per genotype and condition). Microglial cells are stained with Iba1 and astrocytes with GFAP
