# Supplementary material for: Tauroursodeoxycholic bile acid arrests axonal degeneration by inhibiting the unfolded protein response in X-linked adrenoleukodystrophy
Source: Acta Neuropathol. 2016 Dec 21;133(2):283–301. doi: 10.1007/s00401-016-1655-9 (PMC5250669; doi:10.1007/s00401-016-1655-9)
Supplement: Supplementary file 1 — Supplementary material 1 (DOCX 29 kb) [file 401_2016_1655_MOESM1_ESM.docx]

**Supplemental Experimental Procedures**

**MATERIALS AND METHODS**

**Human samples**

Brain tissue samples from X-ALD patients and age-matched controls were obtained from the NIH NeuroBioBank. Frozen blocks of normal-appearing white matter were dissected from frontal or parietal lobes from controls and CCALD and cAMN patients. All children and adults with cerebral ALD had the more frequent parieto-occipital form of cerebral ALD. White matter sections from X-ALD patients and controls were stained with LFB to detect demyelination, demyelination edge and normal-looking areas. Brain tissue sections were processed when two to three adjacent sections showed no sign of demyelination with LFB staining and no perivascular cuffs of lymphocytes using haematoxylin and eosin staining. Detailed information on the post-mortem tissue samples is summarized in supplemental Table S1.

**Immunofluorescence**

For IF, paraffin-embedded sections were incubated in xylene at RT for 15 min and then transferred sequentially into 100% EtOH, 95% EtOH, 70% EtOH, and 50% EtOH for 4 min at room temperature (RT). Sections were rinsed in deionized water and stored in PBS. Slides were immersed in antigen retrieval buffer (10 mM citrate, pH 6.2), boiled for 20 min, and then allowed to cool to RT. Slides were rinsed in PBS. To eliminate autofluorescence, sections were immersed 5 min in EtOH 70%, incubated in Sudan Black solution for 15 min, washed quickly with EtOH 70% and then rinsed twice with PBS. Slides were transferred to a slide staining rack and blocked for 1 h at room temperature with 10% foetal bovine serum (FBS). Slides were incubated overnight at 4°C with proper dilution of primary antibodies in 1% FBS solution. Slides were then washed twice with PBS and incubated for 1 h at RT with secondary antibodies. After three washes with PBS, the nuclei were stained with DAPI for 1 min (2.5 µg/mL; Invitrogen) and rinsed twice with PBS. Slides were mounted in Fluoromount (Sigma) and stored at 4°C. Images from slides were routinely collected within 0–4 days after labelling.

To quantify GRP78 and PDI levels in neurons and astrocytes, a single in-focus plane was acquired. Using ImageJ, an outline was drawn around each cell and area and the mean fluorescence was measured, along with several adjacent background readings. We then calculated the total corrected cellular fluorescence (TCCF)=integrated density – (area of selected cell × mean fluorescence of background readings), and presented it as the protein expression fold change.

**Immunoblot analysis**

Tissue samples were lysed in ice-cold RIPA buffer (50 mM Tris-HCl, pH 8, 12 mM deoxycholic acid, 150 mM NaCl and 1% NP40, supplemented with Complete Protease Inhibitor Cocktail (Roche) and Phosphatase Inhibitor Cocktail “PhosSTOP EASYpack,(Roche)) using a Teflon-on-glass homogenizer and then centrifuged at 1500 *g* for 10 min at 4°C. The protein concentration was determined using a BCA protein assay kit (Thermo Fisher Scientific, Inc.). Samples were boiled for 5 min in Laemmli’s buffer and run on Bis-tris gels. After electrophoresis, proteins were transferred to nitrocellulose membranes using the iBlot^®^ 2 Dry blotting system (Life Technologies) and then incubated with appropriate antibodies. Proteins were visualized with an enhanced chemiluminescence western blot detection system (GE Healthcare Bio-Sciences AB), followed by exposure to CL-XPosure Film (Thermo Scientific).

Protein Bands were quantified by the densitometry function of the Quantity One software.

**Nuclear fractionation**

For the nuclear extraction, isolated spinal cords were washed with ice-cold PBS and resuspended in the lysis buffer (10mM HEPES pH 7.9; 1,5mM MgCl_2_; 10mM KCl, 2,5 M sucrose, 1 mM dithiothreitol (DTT), supplemented with Complete Protease Inhibitor Cocktail (Roche) and Phosphatase Inhibitor Cocktail “PhosSTOP EASYpack (Roche)). After 15 strokes in a Dounce homogenizer, the extract was kept in ice for 20 min and then centrifuged at 800 *g* for 15 min. The pellet was used to prepare the nuclear fraction and was homogenized with a single stroke in a Dounce homogenizer in the extraction buffer (10 mM HEPES pH 7.4; 1.5 mM MgCl_2_; 0.42 M NaCl; 0.5 mM EDTA; 2.5% (v/v) glycerol; 1  mM dithiothreitol (DTT), supplemented with Complete Protease Inhibitor Cocktail (Roche) and Phosphatase Inhibitor Cocktail “PhosSTOP EASYpack,(Roche)). The suspension was gently shaken at 4°C for 45 min and fractionated at 16 000 *g* for 5 min. The resulting pellet was resuspended in extraction buffer and used as the nuclear fraction. The antibody against PARP1 was used as the control for nuclear extraction.

**Analysis of *XBP1* mRNA processing**

*XBP1* cDNA encompassing the region of the restriction site was amplified by PCR using primers specific for the human (h) or the mouse (m) form of XBP1: forward hXBP1. (**^5’^**AAACAGAGTAGCAGCTCAGACTGC**^3'^)** or forward mXBP1 (**^5’^**AAACAGAGTAGCAGCGCAGACTGC**^3’^**) and reverse m/hXBP1 (**^5’^**TCCTTCTGGGTAGACCTCTGGGAG**^3'^**). The amplicon of the unspliced (474 bp) (*XBP1u* cDNA) and spliced form of *XBP1* cDNA (448 bp) (*XBP1s* cDNA) present 26 bp of length difference. In some cases, half of the PCR amplification product was incubated for 1 h at 37°C with the PstI restriction enzyme, which cuts only the amplicon of the unspliced form of *XBP1* cDNA in two fragments of 291 bp and 183 bp. Then, all samples were run on a 2.5% agarose gel with SYBER safe. The gels were photographed under UV transillumination.
