# Supplementary figures and images for: Tauroursodeoxycholic bile acid arrests axonal degeneration by inhibiting the unfolded protein response in X-linked adrenoleukodystrophy
Source: Acta Neuropathol. 2016 Dec 21;133(2):283–301. doi: 10.1007/s00401-016-1655-9 (PMC5250669; doi:10.1007/s00401-016-1655-9)

Figure S1

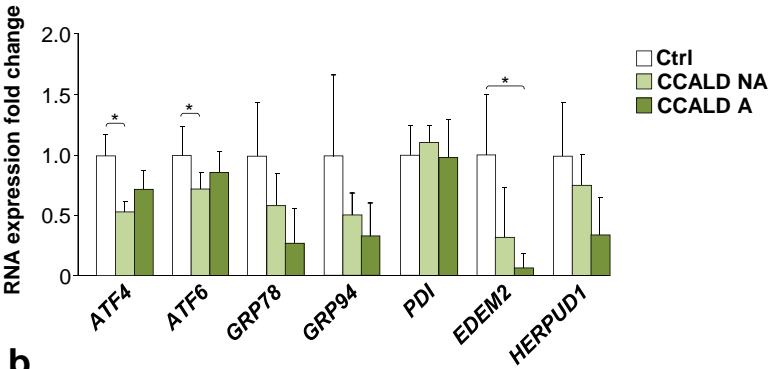

**b**

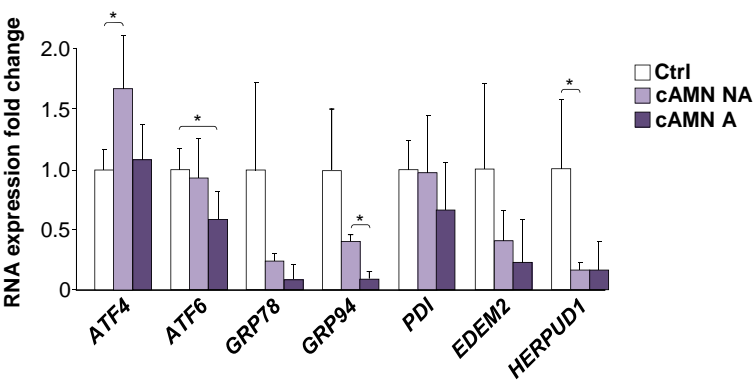

Supplement: Supplementary file 2 — Supplementary material 2 (PDF 56 kb) Fig S1. Downregulation of ATF6 and its targets in affected areas of patient brains. (a-b) Real-time RT-PCR analyses of ATF6, GRP78, GRP94, PDI, EDEM2 and HERPUD1 mRNA in control (Ctrl) samples and in normal-appearing (NA) and affected (A) white matter from (a) CCALD and (b) cAMN patients. Values are expressed as the mean ± SD (n=5 samples per genotype; *P<0.05, **P<0.01 and ***P<0.001, one-way ANOVA followed by Tukey’s HSD post hoc test) [file 401_2016_1655_MOESM2_ESM.pdf]

Figure S2  
**a**

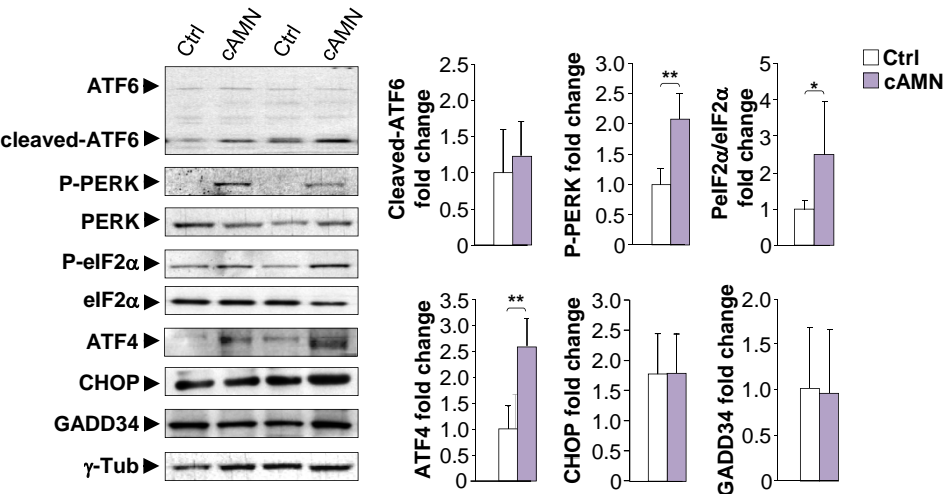

**b**

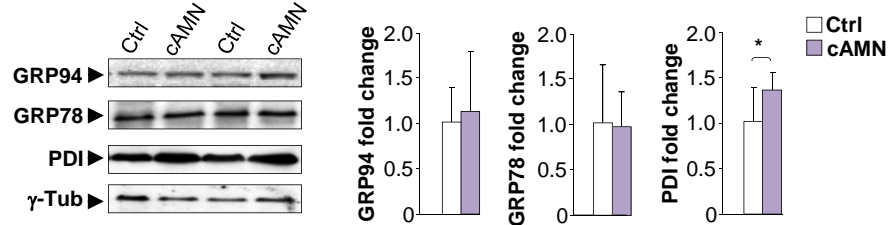

Supplement: Supplementary file 3 — Supplementary material 3 (PDF 145 kb) Fig. S2. Induction of UPR in the spinal cord of cAMN patients. (a) Representative immunoblots for ATF6, PERK, phosphorylated PERK (P-PERK), eIF2α, phosphorylated-eIF2α (P-eIF2α), ATF4, CHOP and GADD34 levels in the spinal cord from control (Ctrl) and cAMN patients. (b) Representative immunoblots for GRP78, GRP94, and PDI levels in the spinal cord from Ctrl and cAMN patients. Protein levels are normalized to γ-tubulin (γ-Tub) levels. The histograms on the right show the ratio and the protein levels relative to control. All values are expressed as the mean ± SD (n=7 by genotype and condition in a-b; *P<0.05 and **P<0. 01, one-way ANOVA followed by Tukey’s HSD post hoc test for a and b) [file 401_2016_1655_MOESM3_ESM.pdf]

Figure S3

a

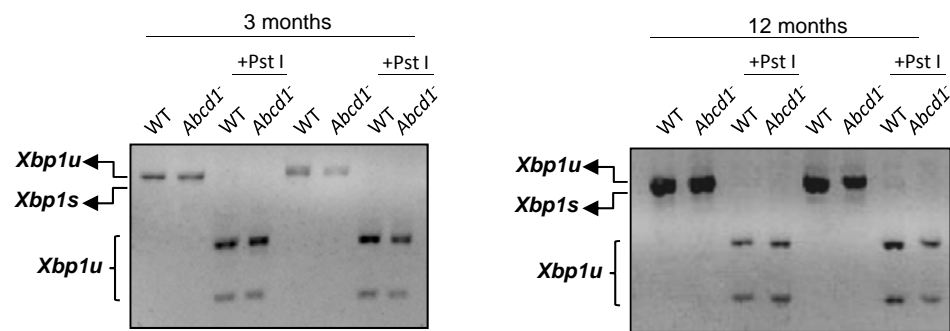

b

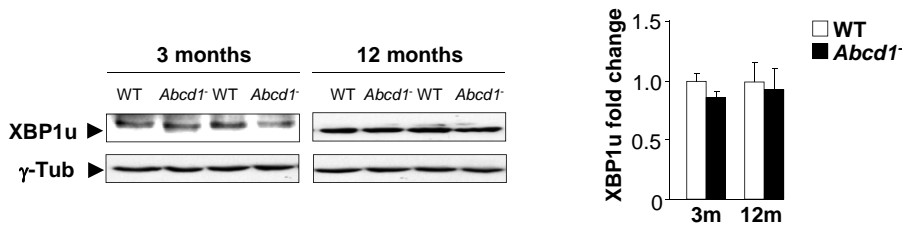

c

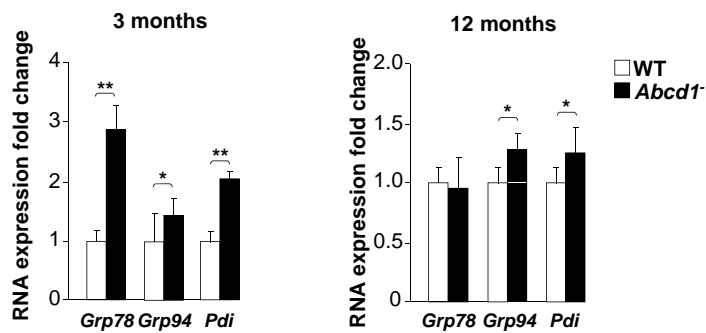

Supplement: Supplementary file 4 — Supplementary material 4 (PDF 122 kb) Fig. S3 (a) Xbp1 cDNA PCR products were cut by PstI (PstI+), producing either two products of 291-bp and 183-bp from the native unspliced form of Xbp1 cDNA; or an uncut product of 448-bp from the spliced Xbp1 cDNA. Unspliced-Xbp1 mRNA: “Xbp1u” mRNA; Spliced-Xbp1 mRNA: “Xbp1s” mRNA. (b) Representative immunoblots of unspliced XBP1 proteins in spinal cords from 3- and 12-month-old Abcd1 - mice and age-matched WT mice. The histogram on the right shows the quantification of XBP1u protein normalized to WT mice. (c) Real-time RT-PCR analyses of Grp78, Grp94 and Pdi at 3 and 12 months in Abcd1 - mouse spinal cords. Values are expressed as the mean ± SD (n= 6 samples per genotype; *P<0.05, **P<0.01 and ***P<0.001, Student’s t test) [file 401_2016_1655_MOESM4_ESM.pdf]

Figure S4  
a

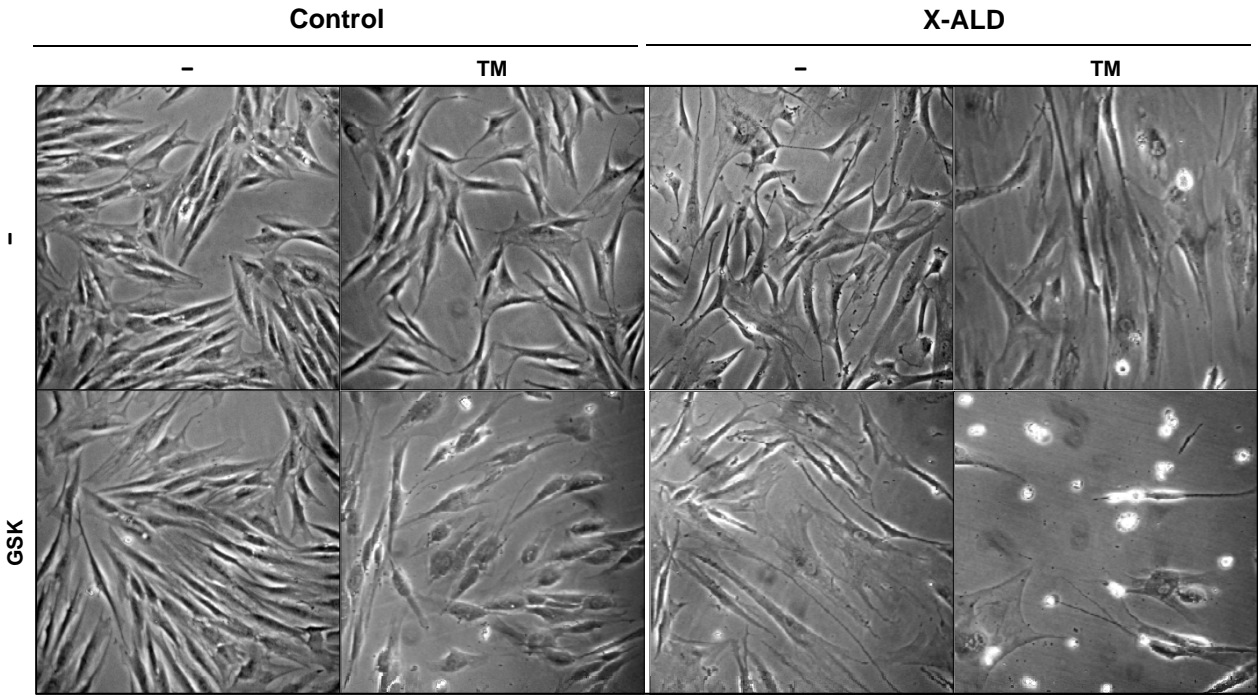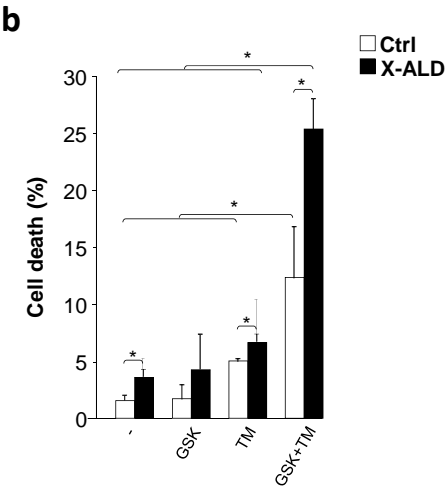

Supplement: Supplementary file 5 — Supplementary material 5 (PDF 723 kb) Fig. S4 X-ALD fibroblasts are more sensitive to tunicamycin upon PERK inhibition. (a) Control and X-ALD fibroblasts were pretreated with or without the PERK inhibitor GSK2606414 (GSK; 120 nM) for 1 h and then exposed to tunicamycin (TM, 2 µg/mL) for 48 h. Pictures of control and X-ALD fibroblasts were obtained (a), and cell death was measured using flow cytometry (b). Values are expressed as the mean ± SD (n=4 by genotype and condition; *P<0.05, **P<0.01 and ***P<0.001, one-way ANOVA followed by Tukey’s HSD post hoc test) [file 401_2016_1655_MOESM5_ESM.pdf]

Figure S5

a

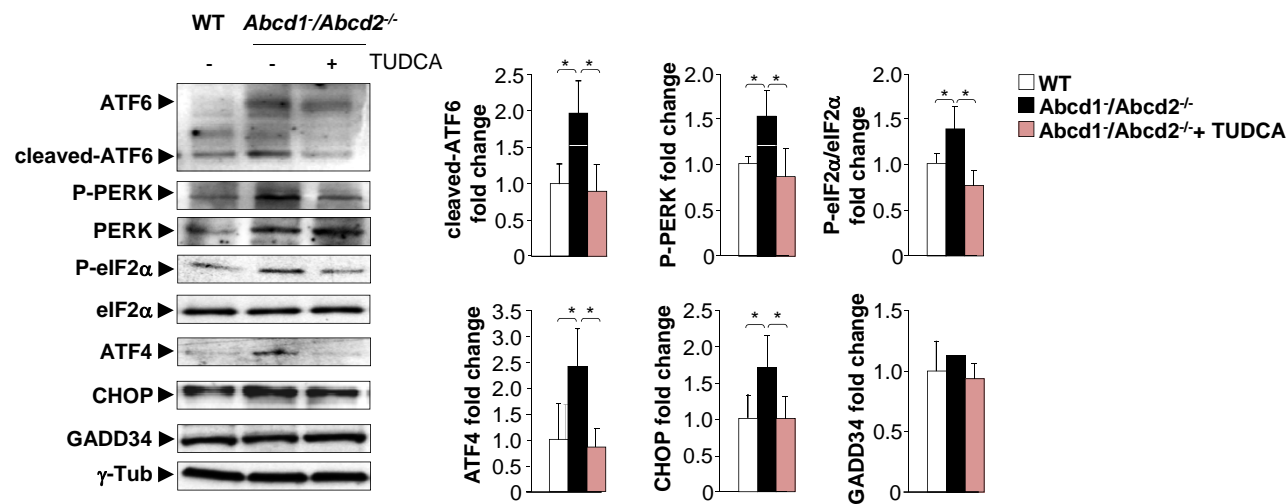

b

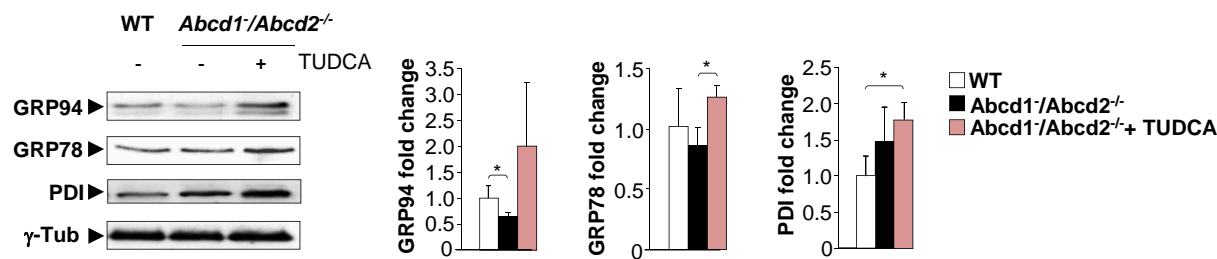

Supplement: Supplementary file 6 — Supplementary material 6 (PDF 162 kb) Fig. S5: UPR induction in the Abcd1 -/Abcd2 -/-mice. (a) Representative immunoblots of ER stress sensors ATF6, cleaved-ATF6, P-PERK/PERK and P-eIF2α/eIF2α ratios, ATF4, CHOP and GADD34 in the spinal cord tissue of wild type (WT), Abcd1 -/Abcd2 -/- and TUDCA-treated (Abcd1 -/Abcd2 -/-+ TUDCA) Abcd1 -/Abcd2 -/-mice 18 months of age. The histograms on the right show the cleaved-ATF6, P-PERK, P-eIF2α, ATF4, CHOP and GADD34 levels normalized relative to γ-Tub and the P-PERK/PERK and P-eIF2α/eIF2α ratios relative to their respective WT values. (c) GRP78, GRP94 and PDI levels were analysed in the spinal cords of WT, Abcd1 -/Abcd2 -/- and Abcd1 -/Abcd2 -/- + TUDCA mice 18 months of age. In (a and b), the histograms on the right show normalized UPR marker levels relative to those in untreated WT mice. All values are expressed as the mean ± SD (n=8 by genotype and condition in a-b; **P<0.01 and ***P<0.001, one-way ANOVA followed by Tukey’s HSD post hoc test) [file 401_2016_1655_MOESM6_ESM.pdf]

Figure S6

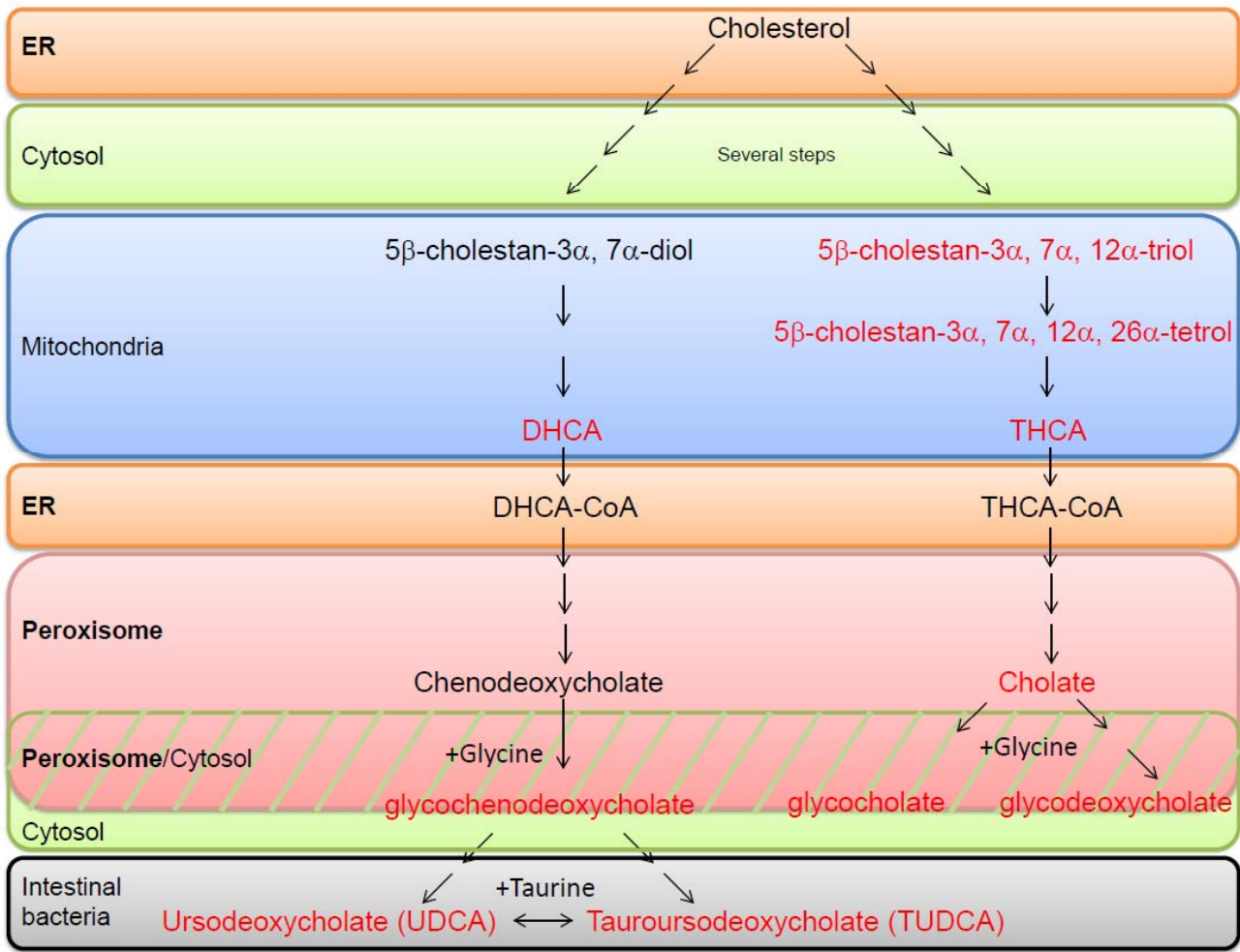

Supplement: Supplementary file 7 — Supplementary material 7 (PDF 144 kb) Fig. S6 Simplified schema of bile acid biosynthesis focused on molecules mentioned in this study and cellular compartments where the biosynthesis is carried out [file 401_2016_1655_MOESM7_ESM.pdf]
