# Supplementary material for: Tauroursodeoxycholic bile acid arrests axonal degeneration by inhibiting the unfolded protein response in X-linked adrenoleukodystrophy
Source: Acta Neuropathol. 2016 Dec 21;133(2):283–301. doi: 10.1007/s00401-016-1655-9 (PMC5250669; doi:10.1007/s00401-016-1655-9)
Supplement: Supplementary file 8 — Supplementary material 8 (PDF 81 kb) Table S1 Description of human brain samples [file 401_2016_1655_MOESM8_ESM.pdf]

Table S1

PHENOTYPE	Sex	AGE	BRAIN WHITE MATTER AREA	Post Mortem Interval (hours)
Control	Male	7	Prefrontal cortex	12
Control	Male	8	Dorsolateral prefrontal cortex	5
Control	Male	11	Dorsolateral prefrontal cortex	16
Control	Male	12	Dorsolateral prefrontal cortex	13
Control	Male	13	Prefrontal cortex	5
Control	Male	36	Dorsolateral prefrontal cortex	18
Control	Male	37	Dorsolateral prefrontal cortex	11
Control	Male	37	Dorsolateral prefrontal cortex	12
Control	Male	37	Dorsolateral prefrontal cortex	9
Control	Male	39	Dorsolateral prefrontal cortex	14
CCALD	Male	9	Dorsolateral prefrontal cortex	15
CCALD	Male	10	Frontopolar prefrontal cortex	1
CCALD	Male	13	Frontopolar prefrontal cortex	14
CCALD	Male	6	Frontopolar prefrontal cortex	12
CCALD	Male	8	Frontopolar prefrontal cortex	1
cAMN	Male	36	Frontopolar prefrontal cortex	2
cAMN	Male	33	Dorsolateral prefrontal cortex	6
cAMN	Male	44	Frontopolar prefrontal cortex	8
cAMN	Male	27	Frontopolar prefrontal cortex	11
cAMN	Male	39	Frontopolar prefrontal cortex	7
