# Supplementary material for: Tauroursodeoxycholic bile acid arrests axonal degeneration by inhibiting the unfolded protein response in X-linked adrenoleukodystrophy
Source: Acta Neuropathol. 2016 Dec 21;133(2):283–301. doi: 10.1007/s00401-016-1655-9 (PMC5250669; doi:10.1007/s00401-016-1655-9)
Supplement: Supplementary file 9 — Supplementary material 9 (PDF 77 kb) Table S2 Description of human X-ALD fibroblasts [file 401_2016_1655_MOESM9_ESM.pdf]

Table S2

Identification	Sex	Genotype	Phenotype
X-ALD-1	Male	X-ALD	AMN
X-ALD-2	Male	X-ALD	AMN
X-ALD-3	Male	X-ALD	AMN
X-ALD-4	Male	X-ALD	AMN
CTL-1	Male		CTL
CTL-2	Male		CTL
CTL-3	Male		CTL
CTL-4	Male		CTL
