# Supplementary material for: Tauroursodeoxycholic bile acid arrests axonal degeneration by inhibiting the unfolded protein response in X-linked adrenoleukodystrophy
Source: Acta Neuropathol. 2016 Dec 21;133(2):283–301. doi: 10.1007/s00401-016-1655-9 (PMC5250669; doi:10.1007/s00401-016-1655-9)
Supplement: Supplementary file 10 — Supplementary material 10 (PDF 107 kb) Table S3 Scaled score corresponding to hindlimb clasping behaviour [file 401_2016_1655_MOESM10_ESM.pdf]

Table S3

SCORE	PHENOTYPE
**0**	Paralysis of the hindlimbs
**0.5**	Both hindlimbs are entirely retracted and touching the abdomen during 100% of the time suspended
**0.75**	Both hindlimbs are entirely retracted toward the abdomen during 100% of the time suspended
**1**	Both hindlimbs are partially retracted toward the abdomen during 100% of the time suspended
**1.25**	alternating clasping and flexion of hindlimbs, although clasping occurs for more than 50% of the time suspended
**1.5**	Alternating clasping and flexion, equally occurring during 100% of the time suspended
**1.75**	Alternating clasping and flexion of hindlimbs, although flexion occurs for more than 50% of the time suspended
**2**	Flexion of hindlimbs during 100% of the time suspended
**2.25**	Alternating flexion and extension of hindlimbs, although flexion occurs for more than 50% of the time suspended
**2.5**	Alternating flexion and extension, equally occurring during 100% of the time suspended
**2.75**	Alternating flexion and extension of hindlimbs, although extension occurs for more than 50% of the time suspended
**3**	Extension of hindlimbs in an angle <90º during 100% of the time suspended
**3.5**	Alternating extension of hindlimbs in an angle <90° and ≥90° during 100% of the time suspended
