# Supplementary material for: Tauroursodeoxycholic bile acid arrests axonal degeneration by inhibiting the unfolded protein response in X-linked adrenoleukodystrophy
Source: Acta Neuropathol. 2016 Dec 21;133(2):283–301. doi: 10.1007/s00401-016-1655-9 (PMC5250669; doi:10.1007/s00401-016-1655-9)
Supplement: Supplementary file 11 — Supplementary material 11 (PDF 85 kb) Table S4 List of antibodies [file 401_2016_1655_MOESM11_ESM.pdf]

Table S4

Antibody	supplier	product number	host	clonality	working dilution
Aldolase A	Novus biologicals	NB600-915	goat	polyclonal	1/1000
APP	Serotec	AHP538	rabbit	polyclonal	1/400
ATF4 (D4B8)	Cell signaling technology	11815	rabbit	monoclonal	1/500
ATF6	Prosci-inc	3683	mouse	monoclonal	1/1000
eIF2α	Cell signaling technology	9722	rabbit	polyclonal	1/1000
GADD153/CHOP	Santa-cruz	sc-793	rabbit	polyclonal	1/1000
GADD34	Santa-cruz	sc-8327	rabbit	polyclonal	1/1000
GFAP	Sigma	G3893	mouse	monoclonal	1/400
GRP78	Sigma	G8918	rabbit	polyclonal	1/1000
GRP94	Abcam	ab63469	mouse	monoclonal	1/1000
Iba1	Wako	019-19741	rabbit	polyclonal	1/1000
IRE1α	Novus biologicals	NB100-2323	rabbit	polyclonal	1/1000
PARP1	Calbiochem	AM30	mouse	monoclonal	1/1000
PDI (RL90)	Abcam	ab2792	mouse	monoclonal	1/1000
P-eIF2α	Cell signaling technology	3398	rabbit	monoclonal	1/500
PERK	Cell signaling technology	3192	rabbit	monoclonal	1/1000
P-PERK	Santa-cruz	sc-32577	rabbit	polyclonal	1/500
Synaptophysin	DakoCytomation	M0776	mouse	monoclonal	1/400
γ-tubulin	Sigma	T6557	mouse	monoclonal	1/2000
Anti-mouse Alexa fluor-488	Invitrogen	A-11001	goat	polyclonal	1/1000
Anti-mouse IgG linked to horseradish peroxidase	Dako Cytomation	P0447	goat	polyclonal	1/5000-1/10000
Anti-rabbit Alexa fluor-555	Invitrogen	A-21428	goat	polyclonal	1/1000
Anti-rabbit IgG linked to horseradish peroxidase	Dako Cytomation	P0448	goat	polyclonal	1/5000-1/10000
