# Supplementary material for: Tauroursodeoxycholic bile acid arrests axonal degeneration by inhibiting the unfolded protein response in X-linked adrenoleukodystrophy
Source: Acta Neuropathol. 2016 Dec 21;133(2):283–301. doi: 10.1007/s00401-016-1655-9 (PMC5250669; doi:10.1007/s00401-016-1655-9)
Supplement: Supplementary file 12 — Supplementary material 12 (PDF 77 kb) Table S5 Summary of the main pathological findings in transversal or longitudinal (1 cm long) sections of the dorsal spinal cord in WT, Abcd1 - /Abcd2 -/-, Abcd1 - /Abcd2 -/- + TUDCA mice at 18m of age (n=5 mice per genotype and condition). Microglial cells are stained with Iba1 and astrocytes with GFAP [file 401_2016_1655_MOESM12_ESM.pdf]

Table S5

| | WT | *Abcd1⁻/Abcd2⁻/⁻* | *Abcd1⁻/Abcd2⁻/⁻* + TUDCA |
|---|---|---|---|
| Iba1 | * | *** | * |
| GFAP | * | *** | * |
| Sudan Black | * | *** | * |
